# Zebrafish *rbm8a* and *magoh* mutants reveal EJC developmental functions and new 3′UTR intron-containing NMD targets

**DOI:** 10.1371/journal.pgen.1008830

**Published:** 2020-06-05

**Authors:** Pooja Gangras, Thomas L. Gallagher, Michael A. Parthun, Zhongxia Yi, Robert D. Patton, Kiel T. Tietz, Natalie C. Deans, Ralf Bundschuh, Sharon L. Amacher, Guramrit Singh

**Affiliations:** 1 Department of Molecular Genetics, The Ohio State University, Ohio, United States of America; 2 Center for RNA Biology, The Ohio State University, Ohio, United States of America; 3 Department of Physics, The Ohio State University, Ohio, United States of America; 4 Department of Chemistry and Biochemistry, The Ohio State University, Ohio, United States of America; 5 Division of Hematology, Department of Internal Medicine, The Ohio State University, Ohio, United States of America; 6 Department of Biological Chemistry and Pharmacology, The Ohio State University, Ohio, United States of America; 7 Center for Muscle Health and Neuromuscular Disorders, The Ohio State University and Nationwide Children’s Hospital, Ohio, United States of America; University of Bern, SWITZERLAND

## Abstract

Many post-transcriptional mechanisms operate via mRNA 3′UTRs to regulate protein expression, and such controls are crucial for development. We show that homozygous mutations in two zebrafish exon junction complex (EJC) core genes *rbm8a* and *magoh* leads to muscle disorganization, neural cell death, and motor neuron outgrowth defects, as well as dysregulation of mRNAs subjected to nonsense-mediated mRNA decay (NMD) due to translation termination ≥ 50 nts upstream of the last exon-exon junction. Intriguingly, we find that EJC-dependent NMD also regulates a subset of transcripts that contain 3′UTR introns (3′UI) < 50 nts downstream of a stop codon. Some transcripts containing such stop codon-proximal 3′UI are also NMD-sensitive in cultured human cells and mouse embryonic stem cells. We identify 167 genes that contain a conserved proximal 3′UI in zebrafish, mouse and humans. *foxo3b* is one such proximal 3′UI-containing gene that is upregulated in zebrafish EJC mutant embryos, at both mRNA and protein levels, and loss of *foxo3b* function in EJC mutant embryos significantly rescues motor axon growth defects. These data are consistent with EJC-dependent NMD regulating *foxo3b* mRNA to control protein expression during zebrafish development. Our work shows that the EJC is critical for normal zebrafish development and suggests that proximal 3′UIs may serve gene regulatory function in vertebrates.

## Author summary

Genetic mechanisms that ensure optimal translation of mRNA into protein are crucial for proper cellular function and normal development. The exon junction complex (EJC) that marks intron-excision sites help achieve optimum mRNA expression by stimulating nonsense-mediated mRNA decay (NMD), which applies brakes on protein production by rapidly degrading mRNAs encoding truncated proteins as well as certain normal mRNAs. A major branch of vertebrate NMD senses mRNAs where stop codons occur ≥ 50 nucleotides upstream of the last intron-excision site and destructs such mRNAs. Our investigation in zebrafish of two EJC proteins Rbm8a and Magoh revealed their critical role during embryonic development, and identified a set of genes where stop codons occur < 50 nucleotides upstream of intron-excision sites, and yet NMD still appears to regulate their mRNA levels. Some mRNAs in human and mouse cells with intron excision events close to stop codons are also apparently regulated by NMD. We identify 167 genes in zebrafish, mouse and human genomes that harbor introns < 50 nucleotides downstream of stop codons and predominantly function in nervous system development. Evidence supports that Rbm8a- and Magoh-mediated suppression of protein production from *foxo3b*, a gene with an intron within 50 nucleotides after its stop codon, is important for zebrafish motor neuron development. Our work shows that the EJC is required for normal development in zebrafish and also indicates that introns close to stop codons may induce NMD to control protein expression. Such mechanisms likely play an important role in gene regulation during vertebrate development.

## Introduction

Post-transcriptional control of messenger RNA (mRNA) expression is critical to regulate location, amount, and duration of protein expression. To achieve optimal protein expression in eukaryotes, many regulatory signals reside in mRNA 3′-untranslated regions (3′UTRs) [1]. Recognition of 3′UTR-embedded signals by RNA binding proteins and miRNAs alter the 3′UTR ribonucleoprotein (RNP) composition and regulate mRNA localization, translation, and stability [1–3]. Nuclear RNA processing steps such as alternative polyadenylation can further impact 3′UTR RNP composition by altering 3′UTR length, and hence the repertoire of 3′UTR regulatory signals [4,5]. Mechanisms dictating 3′UTR RNP composition are thus important for cellular function and organismal development [1,6,7].

Pre-mRNA splicing also greatly impacts RNP composition by imprinting several proteins including the exon junction complex (EJC) on spliced exons [8–10]. The EJC is comprised of three core proteins, Eif4a3, Rbm8a (Y14), and Magoh, which assemble ~24 nt upstream of exon-exon junctions and regulate many post-transcriptional steps including pre-mRNA splicing, mRNA export, localization, translation and nonsense-mediated mRNA decay (NMD) [11–13]. As introns primarily occur in open reading frames and rarely in 3′UTRs, the EJC mainly decorates the translated portion of mRNAs [14], from where they are removed by the first translating ribosome [15,16]. However, if a ribosome terminates translation ≥ 50 nucleotides (nts) upstream of an exon-exon junction, one or more EJCs that remain on the mRNA are now located within the 3′UTR. Such EJCs that occur downstream of a terminated ribosome can engage components of the NMD pathway leading to activation of the central NMD factor UPF1 and rapid mRNA turnover [17,18]. In this way, the EJC can induce destruction of aberrant transcripts bearing premature termination codons to suppress expression of truncated polypeptides. When combined with regulated alternative splicing, such EJC-induced NMD can also suppress expression of particular protein isoforms to regulate cellular homeostasis and developmental decisions [19–21]. Normal mRNAs that contain features such as 3′UTR introns (3′UIs) can also acquire EJCs located in the 3′UTRs (and hence within the 3′UTR RNP) [18,22–25]. In the case of such normal transcripts, the ribosome terminates at a normal stop codon after production of at least one full length polypeptide, but due to the presence of a downstream EJC, the transcript can be targeted for decay. Thus, EJC-dependent NMD also acts as a mechanism to fine-tune protein expression as has been shown for *ARC* mRNA at neuronal synapses [24,25]. Interestingly, 3′UI-bearing transcripts are enriched for neuronal and hematopoietic functions [25], and are expressed in tissue-specific patterns [24], suggesting that 3′UIs may play an important role in regulating tissue-specific developmental programs via EJC-dependent NMD. However, the extent of gene regulation via 3′UI-dependent NMD remains largely unexplored, particularly during development.

The EJC core components Rbm8a and Magoh were first discovered in *Drosophila* for their role in germ cell specification and embryo patterning [26,27]. More recently, mutations in human EJC core protein-encoding genes were shown to cause defects in neural, musculoskeletal, and hematopoietic development [28,29]. Developmental defects in neural cell types are also observed in *Xenopus* embryos and mouse models with reduced EJC core protein levels, suggesting conserved and essential EJC neural functions [30–33]. Recent work in mouse models has illuminated an important role for EJC core components in neural precursor cell proliferation during brain development [34–36]. In mice that are conditionally haploinsufficient for any one of the three EJC core components, neural precursor cells exit the cell cycle early and prematurely differentiate, leading to excessive production of neurons, which then undergo p53-dependent apoptosis [30,31,36,37]. These defects lead to impaired cortical development and microcephaly, a phenotype also associated with *RBM8A* and *EIF4A3* mutations in humans [28,29]. While these advances highlight the critical role of EJC during neural development, much remains to be learned about EJC-regulated developmental gene expression programs and how each of the EJC’s many functions contribute to developmental gene regulation.

In this work, we studied EJC developmental functions in zebrafish, a vertebrate model where embryonic tissue formation and morphogenesis are readily observable. We find that zebrafish Rbm8a and Magoh proteins are deposited on mRNAs similarly in zebrafish as in other vertebrate models and have crucial functions in muscle and neural lineages. In *rbm8a* and *magoh* mutant embryos, EJC-dependent NMD is disrupted. Intriguingly, we find that a set of genes containing a stop codon-proximal 3′UI (intron within 50 nts of the stop codon) are upregulated in zebrafish EJC mutant embryos and *upf1* morphants. We show that transcript and protein levels of *foxo3b*, a proximal 3′UI-containing gene, are elevated in the EJC mutants, and its loss from the mutants significantly rescues their motor neuron outgrowth defect. Possibly, EJC-mediated NMD regulates the *foxo3b* transcript to tune its protein output. Proximal 3′UI-containing genes are also widespread in human and mouse genomes, and are similarly upregulated upon disruption of human and mouse NMD pathways. We identify 167 genes that contain a 3′UI at a stop codon-proximal position in zebrafish, mouse and humans. These genes are enriched for genes encoding RNA-binding proteins and proteins involved in nervous system development. Overall, our work uncovers developmental functions of the EJC and possibly a new set of NMD targets with proximal 3′UIs regulated by EJC-dependent NMD.

## Results

### EJC composition and deposition is conserved in zebrafish

**Fig 1:**
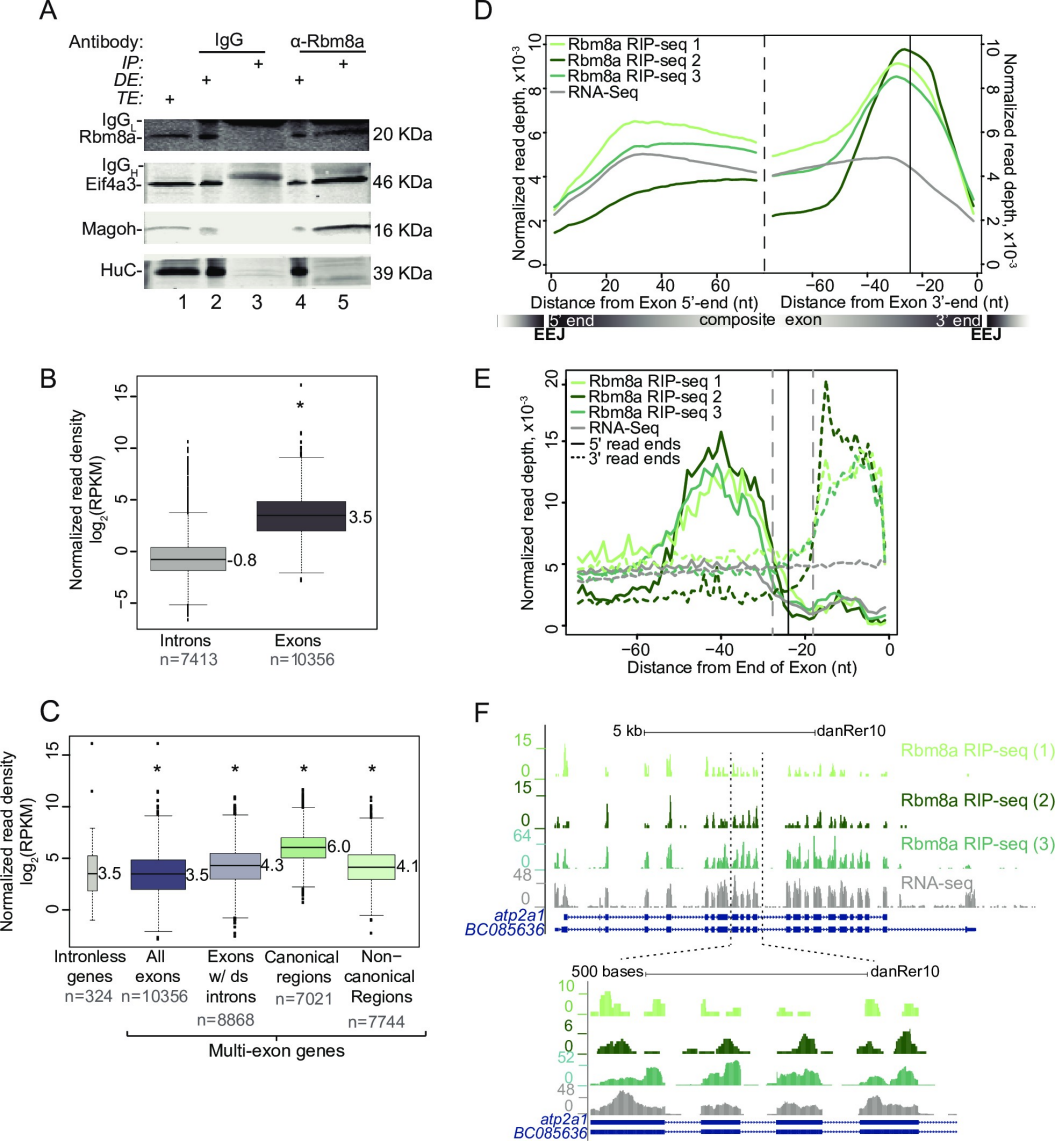
The zebrafish EJC is detected ~24 nucleotides upstream of exon-exon junctions. A. Western blot indicating Rbm8a, Eif4a3, Magoh and HuC proteins detected in RNase I-treated zebrafish embryo total extract (TE, lane 1), depleted extract (DE, lanes 2 and 4) immunoprecipitated protein complexes (IP, lanes 3 and 5). Antigens detected in the blot are listed on the left and antibodies used to immunoprecipitate complexes are listed on top. The signal corresponding to the antibody light chain and heavy chain in the IP lanes is indicated by IgG_L_ and IgG_H_, respectively. B. Boxplots showing the Rbm8a RIP-Seq normalized read densities (reads per kilobase per million, RPKM) in intronic versus exonic genomic regions. Asterisk at the top indicates Wilcoxon test p-value, which is < 10^−6^. C. Boxplots as in B showing the Rbm8a RIP-Seq normalized read densities (RPKM) in the indicated genomic regions (bottom). Exons with downstream introns include all but last exons. Asterisk at the top indicates Wilcoxon test p-values, which are < 10^−6^. D. Meta-exon plots showing Rbm8a RIP-Seq and RNA-Seq (indicated on the top left) normalized read depths in a 75 nt region starting from the exon 5′ (left of dashed black line) or 3′ ends (right of dashed black line). Vertical black line: expected canonical EJC binding site (-24 nt) based on human studies. A composite exon with the relative position of exon-exon junctions (EEJ) is diagrammed at the bottom. E. A meta-exon plot of start and end of Rbm8a RIP-Seq or RNA-Seq reads (indicated on the top left; 5′ ends, solid lines; 3′ ends, dotted lines). Vertical black line: canonical EJC site (-24 nt). Gray vertical dashed lines represent boundaries of the minimal EJC occupied site. F. Top: UCSC genome browser screenshots showing read coverage along the *atp2a1* gene in the Rbm8a RIP-Seq or RNA-Seq replicates as labeled on the right. Bottom: A zoomed in view of the region between the two dotted lines on the top panel. The y-axis on the left of each track shows maximal read coverage in the shown interval.

The three proteins, Eif4a3, Rbm8a, and Magoh, that form the EJC core are highly conserved among multicellular organisms including zebrafish and humans (S1A Fig). To test if the zebrafish EJC core proteins assemble into a complex similar to that observed in human [38–40] and *Drosophila* [41] cells, we immunoprecipitated Rbm8a from RNase-treated zebrafish embryo extracts. We find that both Eif4a3 and Magoh, but not a negative control RNA-binding protein HuC, specifically co-immunopreciptate with Rbm8a (Fig 1A). We and others have previously shown that the EJC primarily binds 24 nts upstream of exon-exon junctions in cultured human cells and adult *Drosophila* [42–46]. To test if the EJC binds at a similar position on zebrafish spliced RNAs, we first optimized RNA-immunoprecipitation (RIP) from RNase-treated zebrafish embryo lysates using an Rbm8a antibody (S1B and S1C Fig). Using optimized conditions, we obtained three well-correlated Rbm8a RIP-Seq biological replicates of Rbm8a-associated RNA fragments from zebrafish embryo lysates (S1D Fig, S1 Table). Rbm8a footprint read densities are significantly higher in exonic regions as compared to intronic regions (Fig 1B), and on exons from multi-exon genes as compared to those from intron-less genes (Fig 1C). A meta-exon analysis shows that, like the human EJC, zebrafish Rbm8a footprints cluster around the canonical EJC position 24 nts upstream of exon 3′ ends (Fig 1D). As expected, 5′ and 3′ ends of RIP-Seq reads accumulate upstream and downstream of the -24 nt position, respectively (Fig 1E). A dramatic reduction in 5′ and 3′ end read counts is seen in a ~10 nt region around the -24 position, revealing the RNA segment that is protected from RNase digestion by Rbm8a-containing EJCs (Fig 1E). The predominant Rbm8a-occupancy position close to exonic 3′ ends is also evident from the RIP-Seq read distribution on individual exons (Fig 1F and S1E Fig). Qualitatively, many canonical EJC sites from highly expressed multi-exon genes show variable Rbm8a binding (Fig 1F and S1E Fig). These profiles also show that zebrafish Rbm8a also associates with non-canonical positions away from the -24 position (Fig 1C, Fig 1F and S1E Fig), as observed previously in human cells [42,43]. Thus, like in human cells, the EJC in zebrafish embryos is also detected at non-canonical positions. Taken together, we conclude that pre-mRNA splicing shapes zebrafish mRNP composition through EJC deposition at exon-exon junctions and beyond.

### *rbm8a* and *magoh* mutant embryos show defects in motility, muscle organization, and motor axon outgrowth

**Fig 2:**
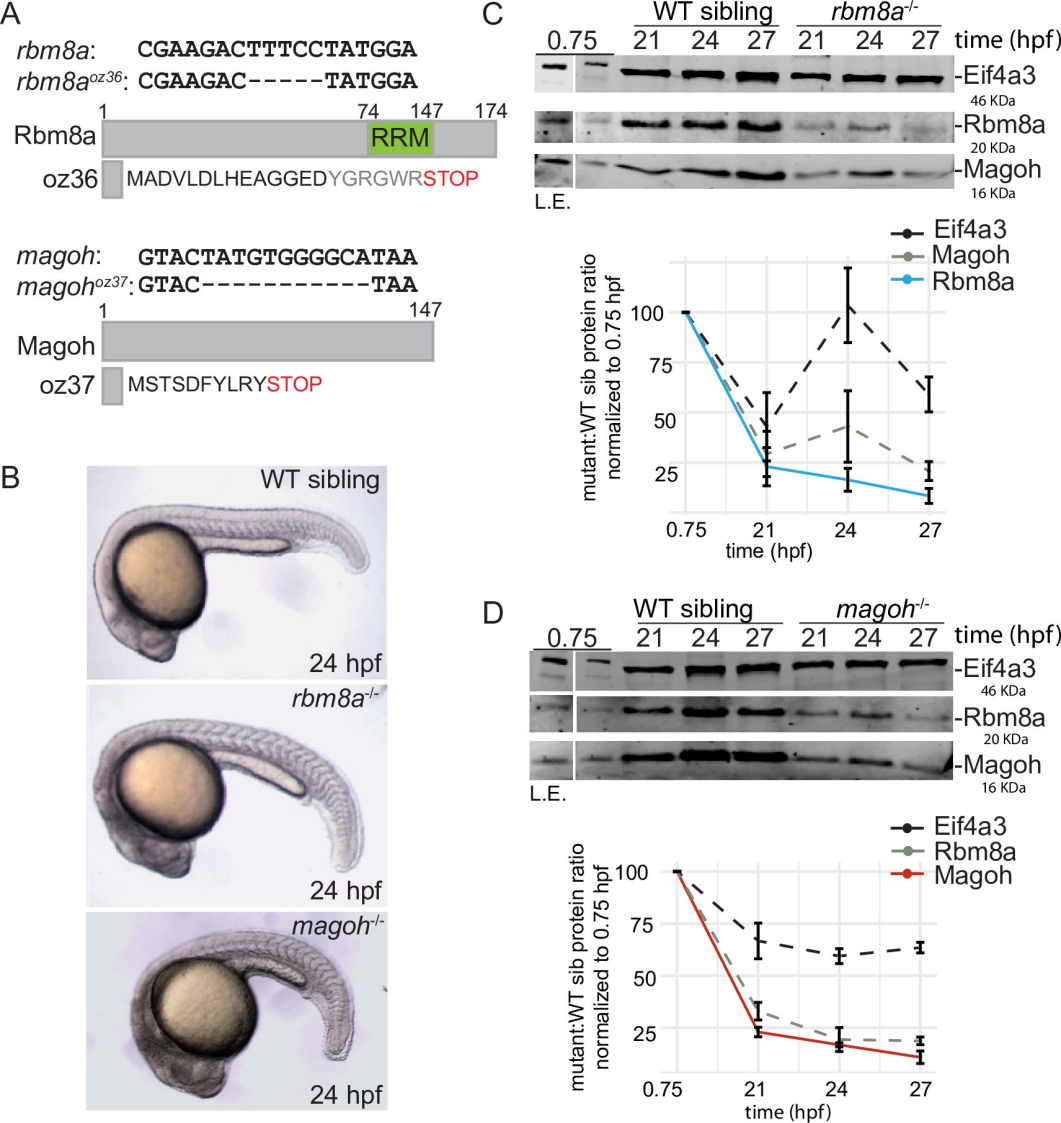
Zebrafish *rbm8a* and *magoh* mutant embryos show gradual loss of maternally contributed Rbm8a and Magoh proteins during early development. A. Schematic illustrating the *rbm8a*^*oz36*^ and *magoh*^*oz37*^ alleles and the predicted proteins they encode. Full-length Rbm8a and Magoh proteins are also shown. RRM: RNA Recognition Motif. B. Whole mount images of live wild-type sibling, *rbm8a* mutant, and *magoh* mutant embryos at 24 hpf. Increased grayness in the head region of homozygous *rbm8a* and *magoh* mutant embryos indicates cell death. C. Top: Western blots showing EJC protein expression in wild type (WT) sibling and *rbm8a*^-/-^ mutant embryos. Antigens detected are listed on the right and embryo genotype is listed above the blot. Developmental time points (hpf) are indicated above each lane. Protein from five (0.75 hpf) or ten embryos (all other time points) was loaded in each lane. A longer exposure (L.E.) of the 0.75 hpf lane is on the left. Bottom: Line graphs showing the amount of protein (per embryo) in the mutant embryos compared to wild-type sibling as a percent of protein present at 0.75 hpf. Error bars represent standard error of means. D. Top: Western blots as in C showing EJC protein expression in wild type (WT) sibling and *magoh*^-/-^ mutant embryos. Bottom: Line graphs showing protein quantification as in C.

To identify the molecular functions of the EJC during embryonic development, we generated zebrafish *rbm8a* and *magoh* mutant embryos. Using a CRISPR/Cas9-based approach [47], we created frame-shifting deletions early in the protein coding sequence to generate null alleles (Fig 2A). Fish heterozygous for *rbm8a*^*oz36*^ or *magoh*^*oz37*^ alleles displayed no obvious phenotypes and were fully viable. Homozygous mutant *rbm8a* or *magoh* embryos (hereafter collectively referred to as EJC mutant embryos), obtained by intercrossing *rbm8a* or *magoh* heterozygotes, initially appear morphologically normal except for head necrosis and tail curvature prior to 24 hpf (hours post fertilization) (Fig 2B). A closer examination revealed that head necrosis is readily detected by acridine orange staining at 19 hpf (S2A Fig) and morphologically by 21 hpf (S2B Fig). After 24 hpf, EJC mutant embryos decline rapidly, with the decline in *magoh* mutant embryos appearing more advanced at each developmental time point examined (Fig 2B, S2B–S2D Fig). Both EJC mutant embryos have reduced head size, pericardial edema, and widespread necrosis by 32 hpf, and die by 48 hpf.

We hypothesized that EJC mutant embryos are initially sustained by maternally-deposited *rbm8a* and *magoh* transcripts [48] and Rbm8a and Magoh protein, and that developmental defects appearing at 19–21 hpf coincide with declining maternal stores. Consistent with maternal deposition of EJC transcript and/or protein, we detect Rbm8a and Magoh protein in 2–4 cell stage embryos (0.75 hpf) (Fig 2C and 2D). By 21 hpf, both Rbm8a and Magoh levels in EJC mutant embryos decrease to ~25% of their respective levels in wild-type siblings, and levels continue to drop over the next six hours (Fig 2C and 2D). As previously observed in mammalian cells [49], reduction of either protein of the Rbm8a:Magoh heterodimer leads to a concomitant depletion of the other protein (Fig 2C and 2D).

**Fig 3:**
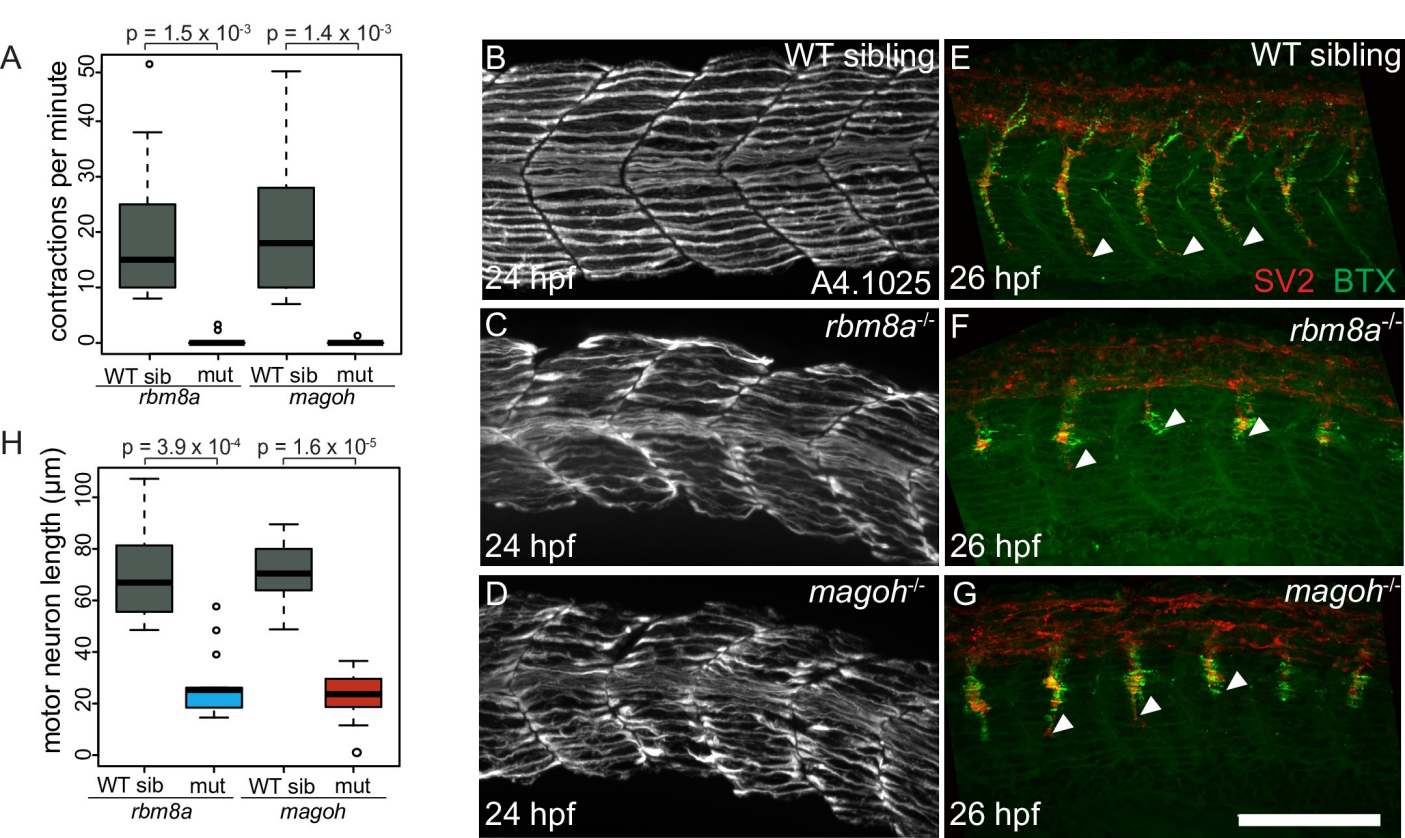
EJC mutant embryos are paralyzed, have disorganized muscles and stunted motor axons. A. Boxplots showing the number of spontaneous contractions per minute measured for the EJC mutant embryos and WT siblings at 24 hpf as indicated on the x-axis. Welch’s t-test p-values are indicated at the top. B-D. Immunofluorescence images showing Myh1 expression in somites 10–14 of WT sibling (B) *rbm8a* mutant (C) and *magoh* (D) mutant embryos. Antibody used was anti-A4.1025 (see methods) (N = 10 embryos/genotype). E-G. Merged confocal images of somites 12–16 in WT siblings (E) *rbm8a* (F) and *magoh* (G) mutant embryos showing immunofluorescence detection of motor neurons (anti-SV2; red) and acetylcholine receptors (α-Bungarotoxin; green). Neuro-muscular junctions in the merged image appear yellow. White arrowheads point to the end of the motor neuron. Scale bar in G is 100 nm. H. Boxplots showing the quantification of motor axon length in somites 12–15 of wild-type sibling, *rbm8a* mutant, and *magoh* mutant embryos (N = 4 embryos/genotype and 4 neurons/embryo). Welch’s t-test p-values are at the top.

Although EJC mutant embryos are morphologically indistinguishable from wild-type siblings at 18 hpf, we find that they are paralyzed (Fig 3A). It is unlikely that the lack of spontaneous contractions is due to developmental delay as EJC mutant embryos progressively worsen and thus, never become motile (S2D Fig). To further characterize the paralysis phenotype, we assessed muscle and motor neuron morphology, as these cell types are required for motility. Myosin heavy chain immunostaining reveals that EJC mutant embryos have disorganized myofibers and have U-shaped instead of chevron-shaped myotomes (Fig 3B–3D), with muscle defects in *magoh* mutant embryos consistently more severe than in *rbm8a* mutant embryos. Co-labeling of motor axons (using anti-SV2) and neuromuscular junctions (using Alexa Fluor-conjugated α-Bungarotoxin), shows that motor axon length and neuromuscular junction number are reduced in EJC mutant embryos (Fig 3E–3H). Thus, as expected of genes that encode proteins that function as a complex, homozygous *rbm8a* and *magoh* mutant embryos show phenotypically similar muscle organization and motor axon outgrowth defects.

### Analysis of gene expression in *rbm8a* and *magoh* mutant embryos

**Fig 4:**
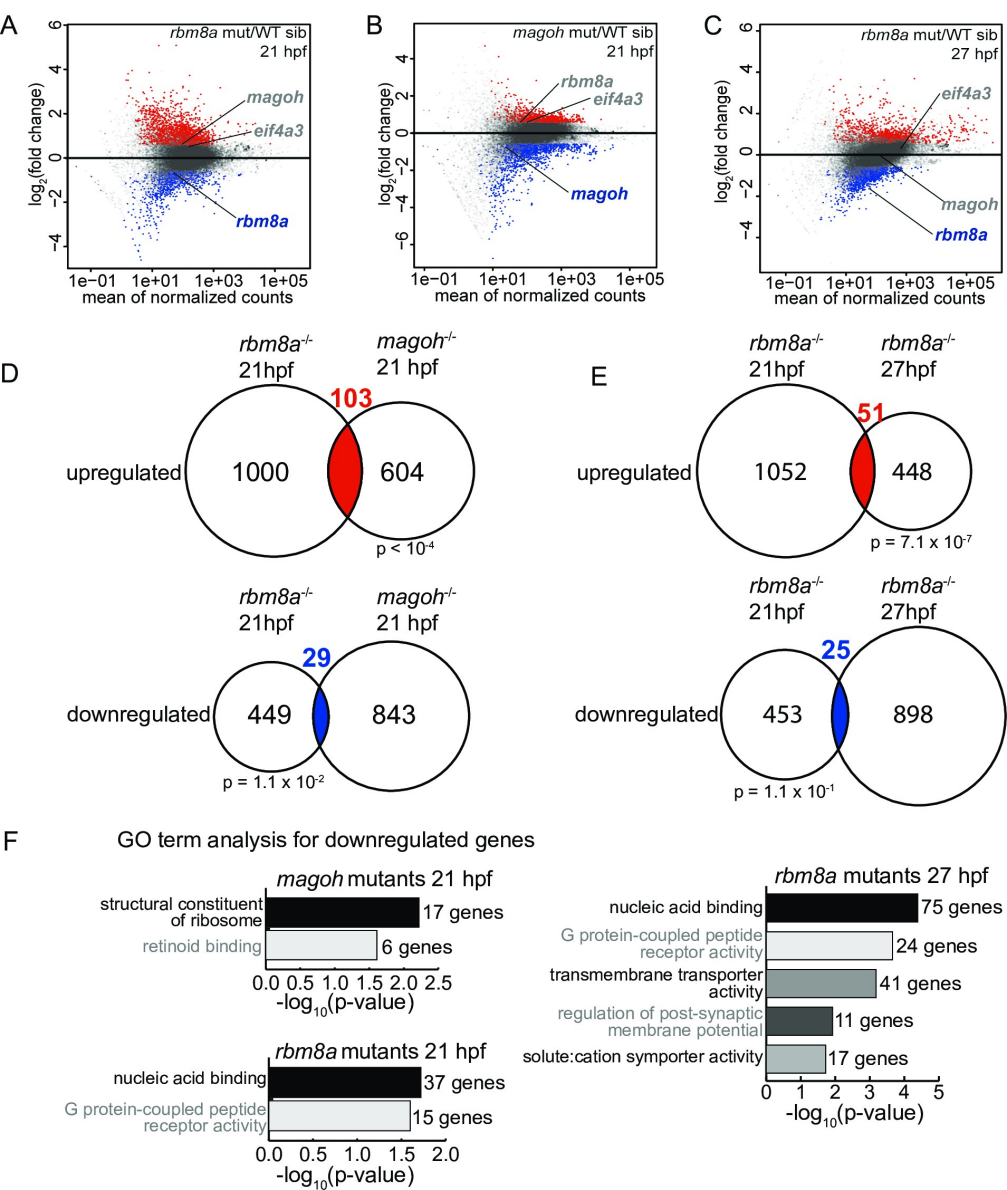
Gene expression changes in *rbm8a* and *magoh* mutant embryos. A-C. MA plots (M: log ratio; A: mean average) showing genes that are upregulated (fold change > 1.5 and FDR < 0.05) (red), downregulated (fold change < 1.5 and FDR < 0.05) (blue), or unchanged (gray) in *rbm8a* mutant embryos compared to WT siblings at 21 hpf (A), *magoh* mutant embryos compared to WT siblings at 21 hpf (B), and *rbm8a* mutant embryos compared to WT siblings at 27 hpf (C). *rbm8a*, *magoh*, and *eif4a3* are labeled in each plot with label colors signifying no change (gray) or downregulation (blue). D. Venn diagrams showing the overlap between genes that are upregulated (top) and downregulated (bottom) in *rbm8a* mutant embryos at 21 hpf (left) and *magoh* mutant embryos at 21 hpf (right). Hypergeometric test p-values are below each comparison. E. Venn diagrams as in (D) comparing upregulated and downregulated genes in *rbm8a* at 21 (left) and 27 hpf (right). F. PANTHER14.0 [86] gene ontology (GO) term overrepresentation analysis of genes downregulated in *rbm8a* and *magoh* mutant embryos at indicated times. All significant terms (Benjamini-Hochberg corrected p-value < 0.05) are shown for each set. The number of genes in each term is indicated at the right of each bar.

To identify EJC-regulated genes during zebrafish embryonic development, we performed RNA-Seq from the EJC mutant embryos and their wild-type siblings at two developmental time points, 21 hpf and 27 hpf. The 21 hpf timepoint is when EJC mutant embryos begin to show visible phenotypes and reduced Rbm8a and Magoh protein levels (Fig 2C and 2D), but do not yet display extensive cell death. By 27 hpf, EJC mutant embryos have reliably low Rbm8a and Magoh protein levels as well as motor axon defects. However, because *magoh* mutant embryos display extensive necrosis at 27 hpf (S2C Fig), we only focused on RNA-Seq from the less necrotic *rbm8a* mutant embryos at this later time point. We generated three biological replicates of total RNA-Seq from each mutant (*rbm8a* mutant at 21 hpf and 27 hpf, and *magoh* mutant at 21 hpf, S1 Table) and their wild-type siblings, a mixture of wild-type and heterozygous embryos. Although the latter two genotypic classes may have differences in their gene expression profiles, we have combined them since heterozygous animals are phenotypically indistinguishable from homozygous wild-type siblings and are fully viable and fertile. A differential gene expression analysis using DESeq2 identified gene-level expression changes in the two mutant embryos compared to their wild-type siblings. As expected, *rbm8a* and *magoh* transcripts are downregulated in the respective mutant embryos (Fig 4A–4C). We compared genes that are significantly altered (fold-change > 1.5, false discovery rate (FDR) < 0.05) among the different mutant embryos and time points. A significant number of genes are up- or down-regulated (Fig 4D, 103 upregulated and 29 downregulated) in both 21 hpf *rbm8a* and *magoh* mutant embryos. Similarly, a significant number of genes are upregulated between *rbm8a* mutant embryos at 21 and 27 hpf (Fig 4E). The modest overlaps observed between differentially expressed genes in EJC mutant embryos could be due to differences in developmental timing or due to variable degree of protein depletion (see discussion).

We next determined if genes with shared functions are enriched among differentially-expressed genes in EJC mutant embryos. Except for a handful of cell death regulators and effectors, none of the upregulated genes in either EJC mutant identify a functionally-related class of genes, suggesting that the proteins they encode perform a variety of functions. The upregulated cell death genes include *tp53*, *tp53-inp1*, and *casp8* (the latter upregulated only in *rbm8a* mutant embryos), which is consistent with cell death observed in mutant embryos (Fig 2B and S2 Fig). In contrast to upregulated genes, downregulated genes in each EJC mutant are enriched in specific GO terms. In *rbm8a* mutant embryos, downregulated genes at both 21 and 27 hpf are significantly enriched for genes encoding proteins with G-protein coupled receptor (GPCR) or nucleic acid binding activities (Fig 4F). In *magoh* mutant embryos at 21 hpf, downregulated genes are significantly enriched for the retinoid binding GO term, which includes several GPCRs. Another functionally-related group of genes downregulated in *magoh* mutant embryos at 21 hpf are genes encoding structural constituents of the ribosome (Fig 4F). This latter class is also downregulated in mouse *magoh* heterozygotes [31], highlighting the importance of *magoh* in ribosomal gene expression during development. Finally, we observe that genes encoding muscle-specific myosins (e.g. *mylpfb*, *myl10*, *myhz2*) and several neural-specific genes (e.g. *rgs17*, *st8sia5*, *lnx2b*, *camk4*) are downregulated in EJC mutants, which may result from altered development and/or loss/reduction of muscle and neuronal cell types (Fig 2 and S2 Fig).

Despite reliable quantification of gene-level differences in expression in the two mutants, surprisingly, we observed only a few changes in splicing patterns in *rbm8a* and *magoh* mutants using the DEX-Seq approach [50], with no overlapping changes between the mutants to report. Possibly, short read lengths (median length ~35 bp) in our RNA-seq data precludes robust quantification of exon-junctions in mutant versus wild-type animals. Therefore, we could not assess whether the EJC functions during pre-mRNA splicing in zebrafish embryos, a role conserved in human, mouse and flies [31,51–53].

### *rbm8a* and *magoh* mutant embryos have defects in NMD

**Fig 5:**
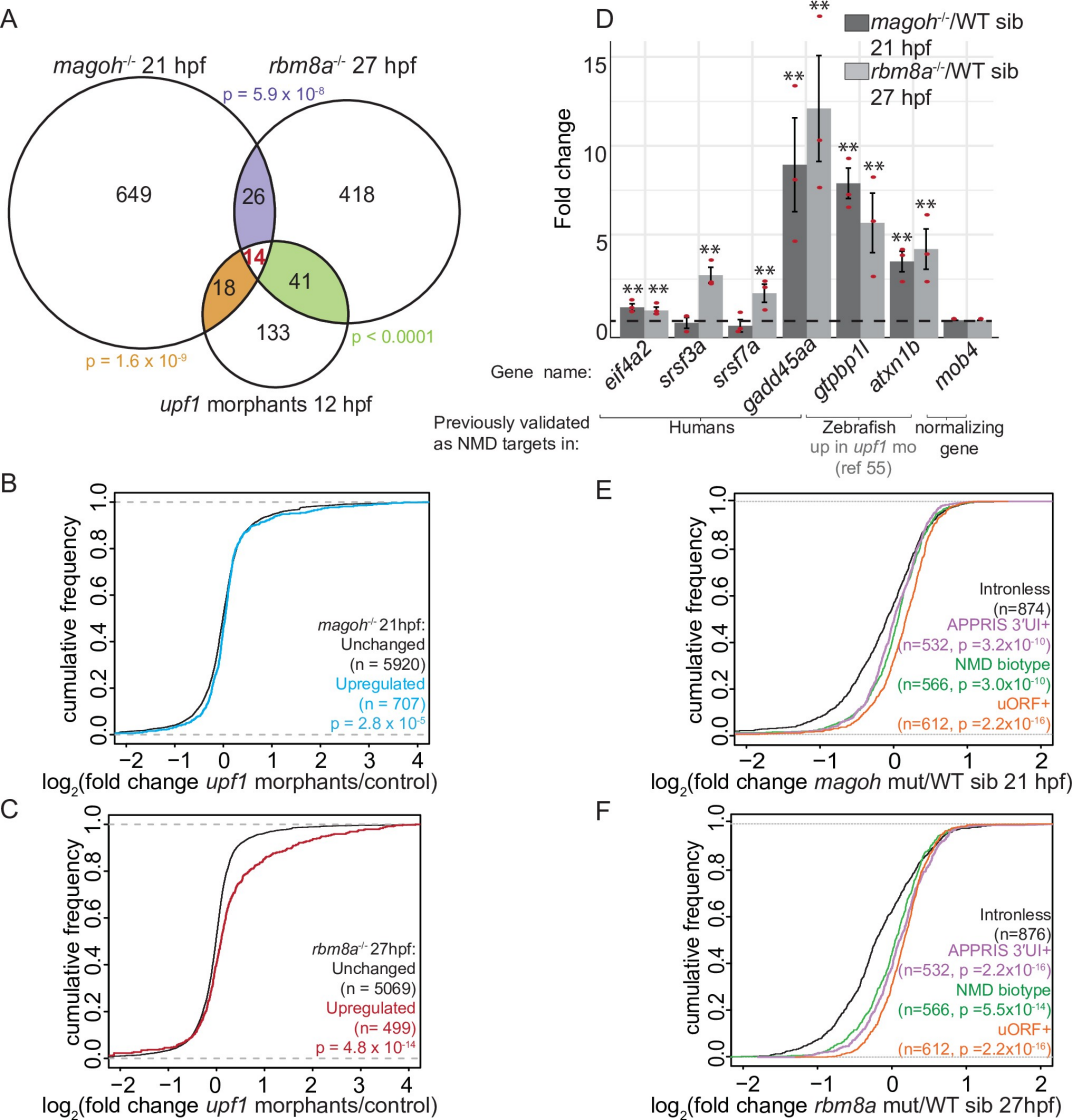
Genes upregulated in EJC mutant embryos are also regulated by Upf1 and contain NMD-inducing features. A. Venn diagram showing the overlap of significantly upregulated genes in EJC mutant embryos and *upf1* morphants. Each overlap and its corresponding hypergeometric test-based p-value are color-coded. B. Cumulative distribution frequency (CDF) plot shows the empirical cumulative distribution of the fold changes in *upf1* morphants (12 hpf) for genes upregulated (blue) and unchanged (black) in *magoh* mutant embryos at 21 hpf. The empirical CDF is the proportion of all values less than or equal to the total number of observations in the group. The CDF function shown here and in all subsequent figures (except Fig 7D) is plotted as an increasing step function along the y-axis with a jump of 1/N at each value of gene number equal to an observed value of fold change (mutant/ WT) (x-axis). Thus, the group of genes that show a rightward shift along the x-axis when compared to control group of genes is a measure of their upregulation in mutants compared to WT siblings (x-axis label). Kolmogorov-Smirnov (KS) test p-value for differences in fold changes between the two groups is indicated on the bottom right. C. CDF plot as in B for genes upregulated in *rbm8a* mutant embryos at 27 hpf (red) compared to unchanged genes (black). D. Quantitative RT-PCR (qRT-PCR) analysis showing fold change of select NMD target transcripts (x-axis) compared to control (*mob4*) transcript in *magoh* mutant embryos at 21 hpf compared to wild-type siblings (dark gray bars) and in *rbm8a* mutant embryos at 27 hpf compared to wild-type siblings (light gray bars). The selected genes either contain a 3′UTR intron (*eif4a2*, *srsf3a* and *srsf7a*) and/or have orthologs that are known NMD targets (*gadd45aa*) or were previously shown to be zebrafish Upf1 targets (*gtpbp1l*, *atxn1b*) [55]. Red dots: the value of each individual replicate. Error bars: standard error of means. Horizontal black dashed line: fold change = 1. Welch’s t-test p-values are indicated by asterisks (** p-value < 0.05). E. Empirical CDF plot, as plotted in B, of fold changes in 21 hpf *magoh* mutant embryos for genes that contain 3′UTR introns (APPRIS 3′UTR intron, mauve), uORF (orange), defined in Ensembl as NMD-biotype (green) compared to intron-less genes (black). KS test p-value for differences in distribution of fold changes between intron-less genes and each of the particular groups is indicated on the bottom right. F. CDF plot as in E showing the fold changes in *rbm8a* mutant embryos at 27 hpf.

Because translation termination upstream of exon-exon junctions was previously shown to trigger NMD in zebrafish embryos [54], one expected group of upregulated transcripts in EJC mutant embryos are mRNAs containing premature termination codons (PTC) or “natural” NMD targets containing a 3′UTR intron (3′UI) or an upstream open reading frame (uORF). To identify whether NMD targets are enriched among upregulated genes in EJC mutant embryos, we first compared genes upregulated in EJC mutant embryos to those upregulated in zebrafish *upf1* morphants at 24 hpf [55]. A statistically-significant number of genes are shared between 24 hpf *upf1* morphants and *magoh* mutant embryos at 21 hpf (39 out of 707, p-value = 1.3 x 10^−2^), and *rbm8a* mutant embryos at 21 hpf (45 out of 1103, p-value < 10^−4^) and at 27 hpf (44 out of 499, p-value = 2.1 x 10^−7^) (S3A Fig). Importantly, NMD targets previously identified in zebrafish *upf1* morphants (e.g. *isg15*, *atxn1b*, *bbc3*) [55] and mRNAs predicted to undergo NMD (e.g. *upb1*, contains a 3′UI) are among these shared genes. We also generated an independent dataset of Upf1-regulated transcripts from zebrafish morphants at an earlier timepoint (12 hpf) using RNA-Seq (in duplicate, S3B Fig, S1 Table) to avoid secondary targets upregulated due to extensive cell death in *upf1* morphants [54]. We find that upregulated genes in 12 hpf *upf1* morphants show a modest but significant overlap with upregulated genes in the previously published 24 hpf *upf1* morphant dataset (S3C Fig); the overlap also includes three of the five NMD targets previously identified by Longman *et al*. (*isg15*, *atxn1b* and *bbc3*) [55]. Statistically significant overlap is also observed among upregulated genes in 12 hpf *upf1* morphants and 21 hpf *magoh* mutant embryos (32 out of 707, p-value = 1.6 x 10^−9^, Fig 5A, S2 Table), and 27 hpf *rbm8a* mutant embryos (65 out of 499, p-value < 10^−4^, Fig 5A, S2 Table); overlap with 21 hpf *rbm8a* mutant embryos is smaller and insignificant. Because the observed changes in *rbm8a* mutant embryos at 21 hpf were similar to but more modest than in *magoh* and *rbm8a* mutant embryos at 21 hpf and 27 hpf, respectively, we focused all subsequent analyses on the latter two EJC mutant datasets. At least one-third of all genes upregulated >1.5 fold upon *upf1* knockdown (FDR < 0.05) also show a >1.5-fold increase (FDR < 0.05) in either 21 hpf *magoh* or 27 hpf *rbm8a* mutant embryos with 14 genes being significantly upregulated in all three datasets (Fig 5A, S2 Table). Globally, genes upregulated >1.5 fold in EJC mutant embryos (FDR < 0.05), as compared to unchanged genes, also show a positive fold-change in *upf1* morphants at both 12 hpf and 24 hpf (Fig 5B and 5C; S3D–S3G Fig). This observation suggests that a much larger shared set of genes show an increase in abundance upon depletion of the EJC or Upf1 even though only a small set is significantly affected.

To independently validate that predicted EJC-dependent NMD targets are indeed affected in EJC mutant embryos, we quantified relative levels of select transcripts that are orthologous to previously validated human NMD targets (*eif4a2*, *srsf3a*, *srsf7a*, and *gadd45aa*), or are upregulated in zebrafish *upf1* morphants [55] (e.g. *gtpbp1l*, *atxn1b*). All of these transcripts are robustly upregulated in at least one of the EJC mutant backgrounds compared to wild-type siblings, and some (*eif4a2*, *gadd45aa*, *gtpbp1l*, *atxn1b*) are upregulated in both EJC mutant backgrounds (Fig 5D). These data further confirm that the EJC is required for Upf1-mediated downregulation of NMD targets in zebrafish embryos.

We next tested if known classes of NMD targets (e.g. PTC-, 3′UI-, uORF-containing mRNAs) are upregulated in EJC mutant embryos. In the Ensembl database, transcripts that contain at least one exon-exon junction > 50 nts downstream of stop codon are flagged as ‘NMD biotype’, and these PTC-containing mRNAs are expected to undergo EJC-dependent NMD. Of the genes encoding transcripts annotated as NMD biotype, 566 genes are detected in our datasets and are upregulated as a group in EJC mutant embryos as compared to a control group of intron-less protein-coding genes (Fig 5E and 5F). We next evaluated features known to induce EJC-dependent NMD of transcripts encoding full length proteins (e.g. 3′UI, uORFs). We limited our analysis to transcripts that are well-supported to encode functional proteins as per the APPRIS database [56]. This genome annotation resource provides manually-curated transcript annotations for zebrafish (and other organisms) where transcripts are classified as principal or alternative isoforms based on conservation, structure and function of each transcript and their encoded proteins. We identified 582 genes that encode APPRIS-annotated transcript isoforms with 3′UIs > 50 nts from stop codons (14 of these are also labeled as ‘NMD biotype’ in Ensembl). Of these, 532 genes that are detected in our datasets are upregulated as a group in EJC mutant embryos when compared to intron-less protein-coding genes (Fig 5E and 5F). Consistent with direct regulation of 3′UI-containing transcripts via EJC-dependent NMD, a greater number of APPRIS-supported transcripts containing this feature show a positive fold change in the two *rbm8a* mutant datasets (S3H Fig). uORFs are another feature that subject mRNAs to NMD to regulate protein expression, and this regulation is sensitive to levels of EJC-associated factors [57]. We identified 620 zebrafish genes encoding APPRIS-annotated transcripts that contain uORFs where ribosome footprints can be detected [58,59]. When compared with a control set of intron-less protein-coding genes, a group of 612 detectably expressed genes containing ribosome-occupied uORFs show a significant positive fold change in EJC mutant embryos (Fig 5E and 5F). Additionally, APPRIS-supported uORF-containing transcripts are enriched within genes showing statistically significant fold change > 1 (log_2_FC > 0) in all EJC mutant and *upf1* morphant datasets (S3I Fig). Overall, we conclude that all major modes to trigger EJC-dependent NMD (i.e. PTCs, 3′UIs and uORFs) are active during zebrafish development.

### Some transcripts with stop codon-proximal 3′UTR introns are upregulated upon loss of EJC and Upf1 function

**Fig 6:**
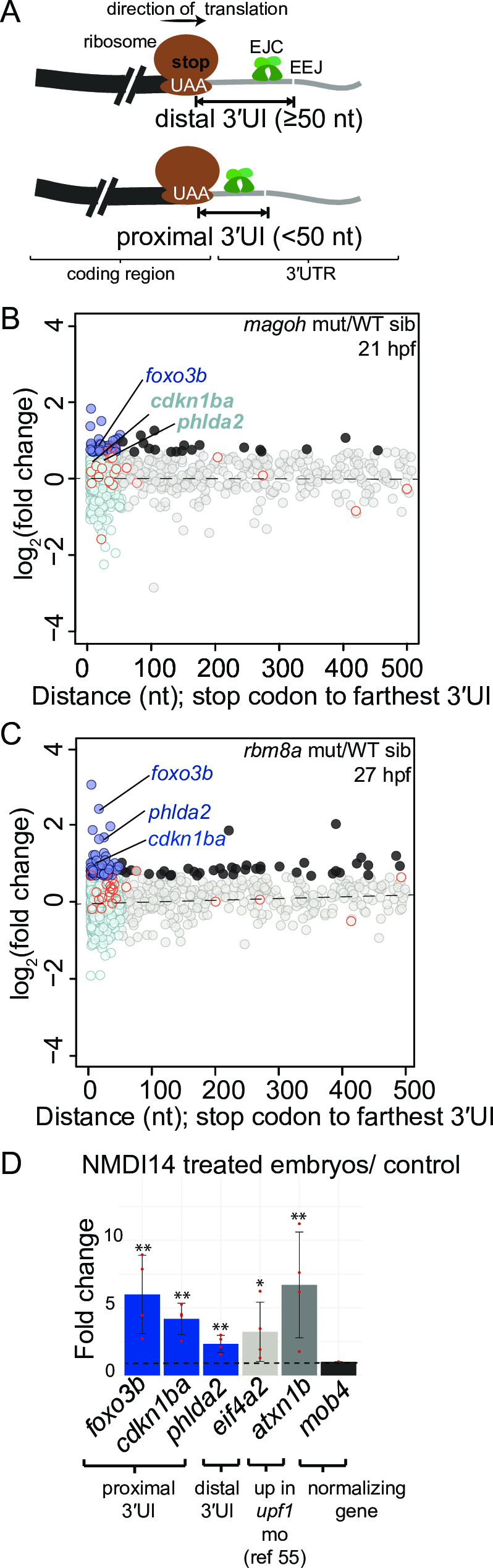
Transcripts encoded by genes with a proximal 3′UTR intron are upregulated in EJC mutant and in NMDI14-treated embryos. A. Top: Schematic illustrating genes with 3′UTR introns (3′UI) where the distance between the stop codon and the 3′UI is equal to or greater than 50 nts. Such 3′UI are classified as distal. Bottom: Schematic illustrating genes with 3′UI where the distance between the stop codon and 3′UI is less than 50 nts. Such 3′UI are classified as proximal. The ribosome (brown), direction of translation (black arrow), stop codon (‘UAA’ in white), EJC (green), exon-exon junction (EEJ), coding region of mRNA (black) and 3′UI of mRNA (gray) are labeled in the top panel. B. A scatter plot showing gene-level fold change (FC) for transcripts with proximal 3′UI (dark blue: FC > 1.5 and light blue: FC < 1.5) and distal 3′UI (black: FC > 1.5 and gray: FC < 1.5) in *magoh* mutant embryos at 21 hpf compared to wild-type siblings. Dots encircled in red represent genes that also contain an upstream open reading frame (see methods). Genes labelled on the plot also contain a proximal 3′UI in mouse and human, and are upregulated in both *rbm8a* mutant datasets (Fig 6C and S4C Fig) and the *upf1* KD dataset (S4D Fig). These genes were independently validated in (D). C. A scatter plot as in B showing fold changes for *rbm8a* mutant embryos at 27 hpf compared to wild-type siblings. D. qRT-PCR analysis showing fold changes for proximal 3′UI-containing genes (blue bars), a distal 3′UI-containing gene (light gray bar), and a Upf1-regulated gene ([55], dark gray bar) compared to the control gene (black bar) in zebrafish embryos treated with NMDI14 from 3–24 hpf. Red dots: the value of each individual replicate. Error bars: standard error of means. Horizontal black dotted line: fold change = 1. Welch’s t-test p-values (** p-value < 0.05; * p-value < 0.1).

Surprisingly, we noticed that among the 14 transcripts that are upregulated in *rbm8a* and *magoh* mutant embryos, and *upf1* morphants (Fig 5A), three (*foxo3b*, *phlda3* and *nupr1a*) are encoded by genes that contain a 3′UI where the distance between the stop codon and the intron is less than 50 nts. For *foxo3b* and *nupr1a*, the human orthologs also contain a 3′UI < 50 nts downstream of the stop codon. This observation raises an intriguing possibility that some mRNAs with a proximal 3′UI (< 50 nts distance between intron and upstream stop codon; Fig 6A) may be regulated by EJC-dependent NMD. Using APPRIS transcript annotations available within Ensembl GRCz10 database, we identified 861 zebrafish genes that encode protein-coding transcripts with proximal 3′UIs; as noted above, 582 genes encode transcripts with distal 3′UIs (3′UIs ≥ 50 nts downstream of the stop codon) (Fig 6A and S4A Fig, S3 Table). Interestingly, proximal 3′UI-containing genes encode proteins which are enriched for mRNA binding and mRNA splicing factor functions (S4B Fig), two functional groups that are well-recognized to be regulated by EJC-dependent NMD [19,60]. We find that, of all proximal 3′UI-containing genes detectable in our datasets, a small percentage (3.5–8%, 70/854 in 27 hpf *rbm8a* mutants, 60/854 in 21 hpf *magoh* mutants and 21/597 in 12 hpf *upf1* morphants), are ≥ 1.5 fold upregulated in EJC mutant embryos (Fig 6B and 6C and S4C Fig) and in *upf1* morphants (S4D Fig). To further confirm that some proximal 3′UI-containing genes are indeed regulated by NMD, we focused on a subset (*foxo3b*, *cdkn1ba*, and *phlda2*) that is upregulated in *upf1* morphants and at least one EJC mutant, and where the existence of a proximal 3′UI is conserved in several other vertebrates including humans. Importantly, these genes show no evidence of additional splicing events in their 3′UTR (S4E Fig). After treating embryos with the NMD inhibitor NMDI14 [61], we found that *foxo3b*, *cdkn1ba*, and *phlda2* transcripts are 2-to-8 fold upregulated, just like *eif4a2*, a distal 3′UI-containing transcript, and *atxn1b*, a previously validated [55] NMD target (Fig 6D). Thus, mRNAs encoded by a subset of genes with a 3′UI in a stop codon-proximal position appear to be regulated in an EJC- and Upf1-dependent fashion. Interestingly, we did not observe any correlation between the distance of the 3′UI from the stop codon and the degree of fold change observed in EJC mutant or *upf1* morphants (Fig 6B and 6C, S4C and S4D Fig, S4F Fig) except for *rbm8a* mutants at 27 hpf where distal 3′UI-containing transcript group shows a higher fold change as compared to the proximal 3′UI-containing group (S4G Fig).

### A subset of human and mouse proximal 3′UI-containing genes may also be regulated by NMD

**Fig 7:**
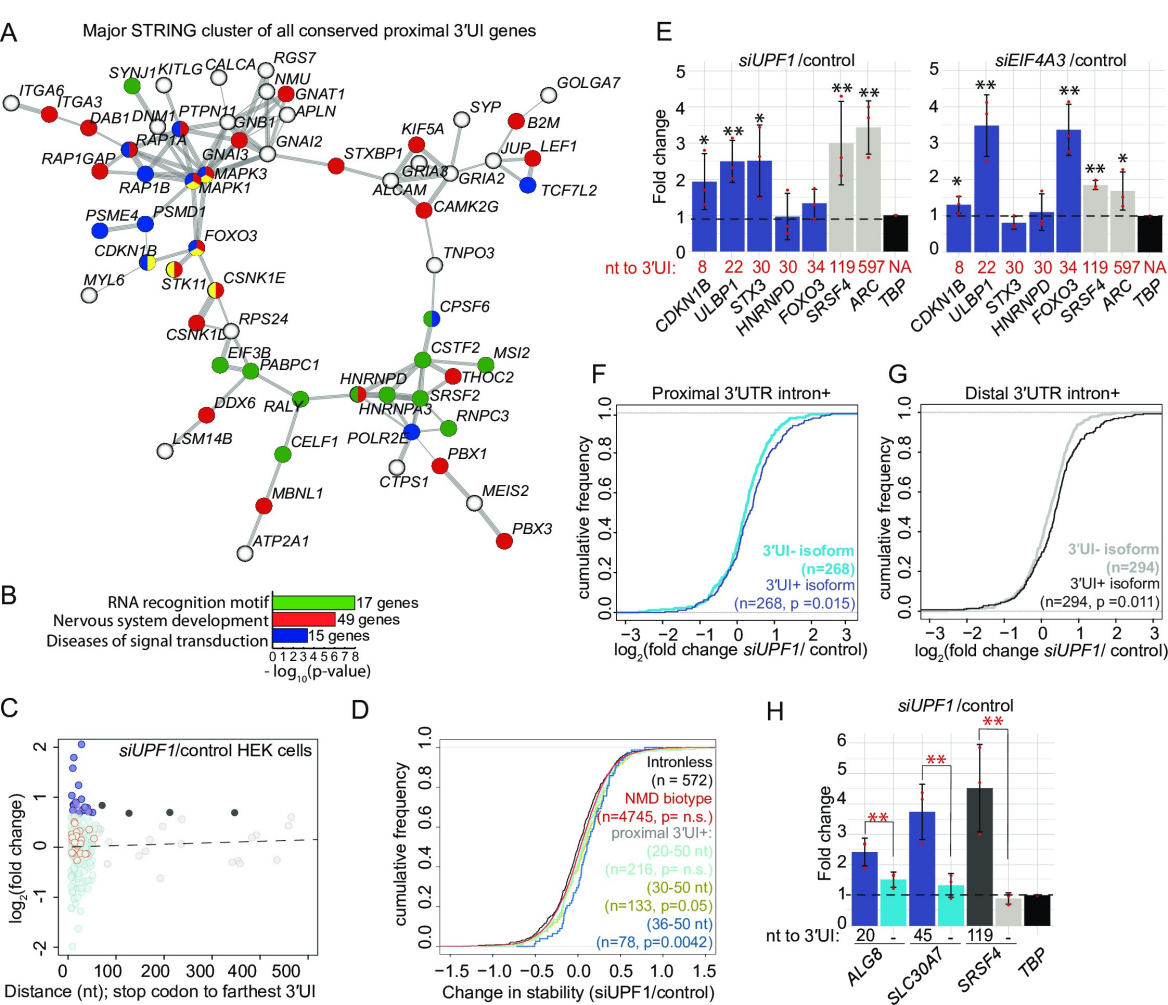
Proximal position of 3′UTR introns is conserved in many vertebrate genes and a subset of proximal 3′UI-containing genes are NMD-sensitive in human cells. A. A major interaction cluster predicted by STRING network analysis of genes with a 3′UI in proximal position in zebrafish, mouse and human. Nodes are colored by gene/protein function: nervous system (red), presence of RNA recognition motif (RRM) (green), diseases of signal transduction (blue), FoxO signaling pathway (yellow). (167 nodes and 127 edges in total, PPI enrichment p-value = 0.02). B. Gene ontology enrichment analysis of all 167 genes with conserved 3′UI proximal positioning. The most significant GO term within the following functional categories are shown: Interpro domains, Biological process and Reactome pathways. C. A scatter plot showing fold changes for APPRIS-annotated proximal 3′UI transcripts (dark blue: FC > 1.5 and light blue: FC < 1.5) and distal 3′UI transcripts (black: FC > 1.5 and gray: FC < 1.5) in *UPF1* knockdown HEK293 cells compared to control cells [57]. Dots encircled in red are transcripts that also contain an uORF as determined previously in [57]. Same analysis for Ensembl transcript annotations is shown in Fig S5G. D. Empirical CDF plot showing change in mRNA stability for different classes of NMD targets and intron-less genes upon *UPF1* knockdown in HEK293 cells (data from [57]). The gene classes are as follows: proximal 3′UI-containing genes where distance is 20–50 nts (light green), 30–50 nts (olive green) and 36–50 nts (dark blue), Ensembl-annotated NMD-biotype genes (red) and intron-less genes (black). CDF function is plotted as in Fig 5B. KS test p-value for comparison of NMD targets to intron-less genes is indicated in the same color. E. qRT-PCR analysis showing fold changes for proximal 3′UI-containing genes *CDKN1B*, *FOXO3* (upregulated in zebrafish); *ULBP1*; *STX3* (highest change in stability upon *UPF1* KD in HEK293 cells [57]); *HNRNPD* (encodes RRM-containing protein) and distal 3′UI-containing genes (*ARC* and *SRSF4*) upon *UPF1* (left) and *EIF4A3* (right) knockdown in HCT116 cells. The distance between stop codon and 3′UI for every 3′UI-containing gene is indicated below each bar. *TBP* is the normalizing gene used for qRT-PCR analysis. Welch’s t-test p-values are indicated using asterisks (** p-value < 0.05 and * p-value < 0.1). F. Empirical CDF plot of fold changes in levels of 3′UI-containing isoforms (dark blue) as compared to 3′UI-lacking isoforms (sky blue) encoded from same genes in *UPF1-*depleted HEK293 cells. KS test p-value for differences in the two distributions is indicated on the bottom right. G. CDF plot as in F showing the fold changes in levels of distal 3′UI-containing (black) versus 3′UI-lacking isoforms (gray). H. qRT-PCR analysis showing fold changes for 3′UI-containing and 3′UI-lacking isoforms encoded by proximal 3′UI-containing genes *ALG8* and *SLC30A7* and by a distal 3′UI-containing gene *SRSF4* upon *UPF1* knockdown in HCT116 cells. The distance of 3′UI when present, or its absence (-) is indicated below each bar. *TBP* is the normalizing gene used for qRT-PCR analysis. Welch’s t-test p-values for the three biological replicates of each transcript are < 0.05. The Welch’s paired t-test p-values for the comparison between fold changes of 3′UI-containing and 3′UI-lacking isoforms is indicated using red asterisks (** p-value < 0.05).

We surveyed human and mouse genomes for prevalence and conservation of proximal 3′UIs. Like in zebrafish, APPRIS-annotated proximal 3′UI-containing genes outnumber distal 3′UI-containing genes in human (S5A Fig and S3 Table, 1239 proximal 3′UI-containing genes, 489 distal 3′UI-containing genes) and mouse (S5B Fig and S3 Table, 921 proximal 3′UI-containing genes, 649 distal 3′UI-containing genes). GO terms of human and mouse genes encoding APPRIS-annotated proximal 3′UI-containing transcripts are also enriched for mRNA binding function (S5E and S5F Fig). A cross-comparison of zebrafish, mouse, and human proximal 3′UI-containing genes identified 167 genes where the proximal position of 3′UI is conserved in all three organisms suggesting that proximal 3′UIs could serve regulatory functions. These genes show a significant interaction network amongst themselves (Fig 7A, p-value = 0.02), and are enriched for genes encoding proteins with RNA recognition motifs and with roles in neural development and disease (Fig 7B).

To test if some human and mouse proximal 3′UI-containing genes are also regulated by NMD, we analyzed publicly available RNA-seq datasets of human and mouse cell lines depleted of key NMD factors. We find that a subset of transcripts encoded by proximal and distal 3′UI-containing genes are similarly upregulated in *UPF1*-depleted HEK293 cells (Fig 7C, S5G Fig) [57] and human ESCs (S5H Fig) [62], and in *Smg6*^*-/-*^ knockout mouse ESCs (S5I Fig) [63]. Furthermore, transcript stability of proximal 3′UI-containing genes grouped based on increasing distance from the stop codon (20–50 nts, 30–50 nts, and 36–50 nts) progressively increases upon UPF1 knockdown in HEK293 cells [57] (Fig 7D). Notably, the proximal 3′UI-containing genes where the intron is ≥ 36 nts from the stop codon are the most significantly stabilized. To further test the UPF1 and EJC dependence of proximal 3′UI-containing NMD targets in human cells, we knocked down *UPF1* or *EIF4A3* in a human colorectal carcinoma cell line (HCT116), and assessed levels of a subset of 3′UI-containing transcripts. This subset consists of human orthologs of all three proximal 3′UI-containing genes validated in zebrafish (*FOXO3 CDKN1B*, and *PHLDA2*). We also chose three proximal 3′UI-containing genes that show the highest change in stability upon *UPF1* knockdown in Fig 7D (*STX3*, *ULBP1* and *RBM3*). We also included HNRNPD, an RNA-binding protein-encoding transcript whose principal APPRIS isoform contains a proximal 3′UI. In these transcripts, the proximal position of the 3′UI is conserved (with the exception of *STX3*;. Importantly, primer pairs used for detection of these transcripts unambiguously amplify only the proximal 3′UI-containing isoforms (S4 Table). We find that, similar to transcripts encoded by distal 3′UI-containing genes (*ARC and SRSF4*), *CDKN1B* and *ULBP1* are significantly upregulated upon *EIF4A3* and *UPF1* knockdown (Fig 7E). *STX3* and *FOXO3* are significantly upregulated either upon *UPF1* or *EIF4A3* knockdown but not under both conditions. The proximal 3′UI-containing isoform of *HNRNPD*, on the other hand, remains unchanged in HCT116 cells upon *UPF1* or *EIF4A3* knockdown (Fig 7E). *PHLDA2* and *RBM3* were below detection limits in HCT116 cells and therefore could not be tested. As further validation of the specificity of the proximal 3′UIs to induce NMD, we find that *UPF1* knockdown in HEK293 cells [57] specifically increases levels of the proximal 3′UI-containing isoforms as compared to 3′UI-lacking isoforms produced from the same gene (Fig 7F), similar to the effect observed for transcript isoforms with or without distal 3′UI (Fig 7G). Further, proximal 3′UI-containing isoforms of *ALG8* and *SLC30A7*, two transcripts that show the highest fold-upregulation in Fig 7F, are also specifically upregulated as compared to their 3′UI-lacking isoforms upon *UPF1* depletion in HCT116 cells (Fig 7H). While these data collectively suggest that the presence of a proximal 3′UI may sensitize certain transcripts for NMD, it remains to be tested if a proximal 3′UI is sufficient to induce NMD or if it acts in concert with/via other NMD signals (see Discussion).

### Loss of function of *foxo3b*, a proximal 3′UI-containing gene upregulated in EJC mutant embryos, partially rescues motor axon outgrowth

**Fig 8:**
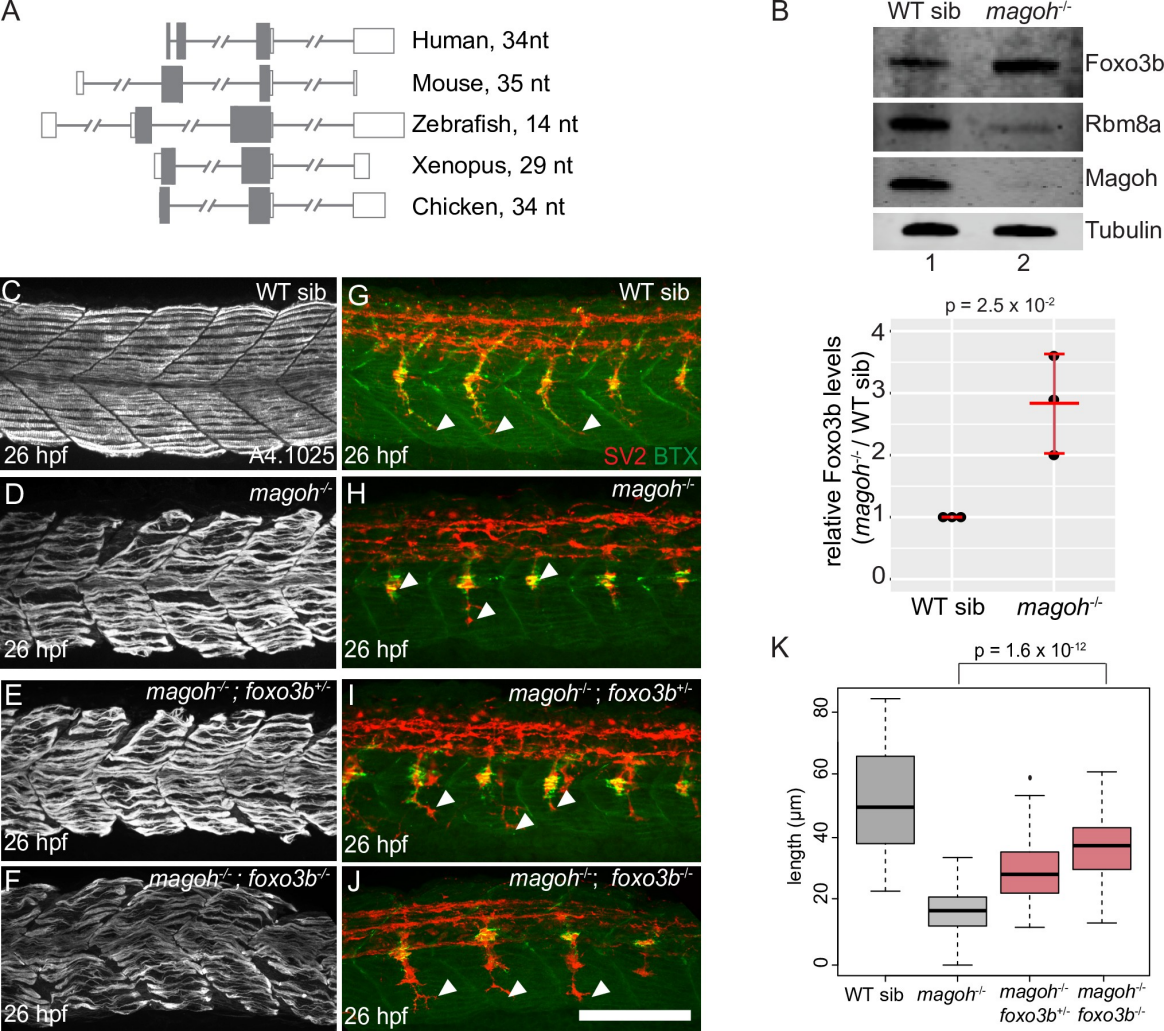
Partial or complete loss of *foxo3b* in *magoh* mutant embryos rescues motor neuron outgrowth defects. A. Illustration showing *foxo3b* gene structure in indicated vertebrates. The distance between the stop codon and the proximal 3′UTR intron is on the right. Open rectangles: UTRs, filled rectangles: coding region, gray lines: introns (hash marks denote shortened intron sequences). B. Top: Western blot showing protein levels in wild-type sibling (lane 1) and *magoh* mutant (lane 2) embryos at 21 hpf. Bottom: a dot plot showing Foxo3b levels normalized to tubulin levels in *magoh* mutant embryos and WT siblings at 21 hpf in three biological replicates. (N = 5 embryos per genotype per replicate). Error bars: standard error of means. C-F. Confocal images showing Myh1 immunofluorescence using anti-A4.1025 in somites 12–16 of WT sibling (C), *magoh*^-/-^ mutant (D), *magoh*^-/-^; *foxo3b*^+/-^ mutant (E), and *magoh*^-/-^; *foxo3b*^-/-^ mutant (F) embryos at 26 hpf (N = 13 embryos/genotype). G-J. Merged confocal images showing motor neurons (red; detected by anti-SV2 staining) and acetylcholine receptors (green; detected by alpha-bungarotoxin staining) in somites 12–16 of WT sibling (G), *magoh*^-/-^ mutant (H), *magoh*^-/-^; *foxo3b*^+/-^ mutant (I), and *magoh*^-/-^; *foxo3b*^-/-^ mutant (J) embryos. Neuromuscular junctions in the merged images are yellow. White arrowheads point to the distal end of the motor neuron. (N = 13 embryos per genotype). Scalebar in J (for panels C-J) is 100 nm. K. Boxplots showing quantification of motor axon length in embryos of genotypes indicated along the x-axis (4 motor neurons/embryo and 13 embryos/genotype). Welch’s t-test p-values for comparison between *magoh*^-/-^ mutant and *magoh*^-/-^; *foxo3b*^-/-^ mutant embryos are at the top.

The zebrafish *foxo3b* gene encodes a transcript that is significantly upregulated in both EJC mutants and *upf1* morphants (Fig 6B and 6C, S4C and S4D Fig and S6A Fig). These data, as well as the conservation of proximal intron position in other vertebrate *foxo3b* homologs (Fig 8A) and the NMD susceptibility of human *FOXO3* (Fig 7E), suggests that *foxo3b* expression is regulated by EJC-dependent NMD. Like *foxo3b* transcript, we find that Foxo3b protein is upregulated in 21 hpf *magoh* mutant embryos (2.8-fold; Fig 8B) and in 27 hpf *rbm8a* mutant embryos (1.3-fold; S6B Fig) compared to wild-type siblings. Furthermore, five known Foxo3b transcriptional target genes [64] are also upregulated in *magoh* and *rbm8a* mutant embryos (S6C Fig). To test if Foxo3b upregulation contributes to EJC mutant phenotypes, we obtained a previously described *foxo3b* null allele, *foxo3b*^*ihb404*^ [65,66], generated *magoh; foxo3b* and *rbm8a; foxo3b* doubly heterozygous adults, and examined muscle and motor neuron development in single, double, and compound mutant embryos. As expected, embryo morphology, motility and motor axon outgrowth of *foxo3b* mutant embryos is indistinguishable from wild-type embryos; hence these mutant embryos are sorted into the wild-type sibling pool. In contrast, as noted above, motor axons barely extend beyond the horizontal myoseptum in EJC mutant embryos (Fig 3E–3G, Fig 8G and 8H and S6H and S6I Fig). Strikingly, we find that heterozygous and homozygous loss of *foxo3b* in EJC mutant embryos leads to significantly longer motor axons that extend well beyond the horizontal myoseptum (Fig 8I–8K and S6J–S6L Fig), but not as far as in wild-type sibling embryos. Despite significant rescue of motor axon outgrowth, neuromuscular junction formation (Fig 8I and 8J and S6J and S6K Fig) and myofiber organization (Fig 8E and 8F and S6F and S6G Fig) are not restored in *magoh; foxo3b* or *rbm8a; foxo3b* double mutant embryos. Thus, we conclude that Foxo3b repression via EJC-dependent NMD is important for motor axon outgrowth. Additionally, we predict that regulation of other mRNA targets is required for proper muscle development.

## Discussion

### Loss of EJC causes tissue-specific defects and embryonic lethality in zebrafish

In zebrafish *rbm8a* and *magoh* single mutant embryos, both Rbm8a and Magoh proteins are co-depleted (Fig 2C and Fig 2D). This simultaneous reduction of *rbm8a* and *magoh* function likely impairs EJC function leading to rapid emergence of developmental defects, which progressively worsen and lead to embryonic death by 2 days post-fertilization. Remarkably, the defects in EJC mutants initially arise in specific tissues. Head necrosis is morphologically apparent in EJC mutants by 19–21 hpf, with onset occurring earlier in *magoh* mutants than in *rbm8a* mutants. This neural cell death phenotype is readily detected in both mutants at 19 hpf with the more sensitive acridine orange dye (S2A Fig). The neural cell death phenotype is similar to that seen in mouse heterozygous EJC mutant embryos [30,31], and is consistent with the microcephaly phenotype in human patients heterozygous for hypomorphic *RBM8A* and *EIF4A3* mutations [28,29]. Thus, across vertebrates, certain tissues appear more sensitive to loss of EJC function. The emergence of defects in EJC mutant embryos in discrete lineages such as neural and muscle cells may result from tissue-specific differences in EJC protein functions, activity of EJC regulators, or decay rates of maternally-provided EJC transcript/protein. Future investigation into these possibilities in zebrafish embryos may explain why loss of a ubiquitously-expressed entity like the EJC leads to tissue-specific phenotypes, as are also observed in human EJC-linked syndromes [28,29]. Notably, unlike haploinsufficiency of EJC core components in mouse and human [28–31], heterozygous loss of *rbm8a* or *magoh* in zebrafish does not have any apparent phenotypic consequences, indicating that the threshold dose of EJC may differ between zebrafish and mammals.

Despite similar phenotypic defects in *rbm8a* and *magoh* mutant embryos, gene expression changes in the two mutants show only a modest (albeit statistically significant) overlap (Fig 4). Multiple factors are likely to contribute to this observation. Foremost, it is likely that at 21 hpf, when gene expression is first compared between *rbm8a* and *magoh* mutants (Fig 4D), the two mutants are at different stages of developing EJC-related defects even though they are at the same developmental time point. In support of this idea, *magoh* mutants show head necrosis at 19 hpf while this phenotype is not readily seen in *rbm8a* mutants until 21 hpf. This temporal difference in appearance of defects in the two mutants may be driven by the timing/rate of depletion of maternal mRNA/protein stores (Fig 2C and 2D). When comparing RNA-seq data sets from *rbm8a* mutants at 21 hpf and 27 hpf (Fig 4E), the six-hour age difference is likely a major factor in the observed gene expression differences. The comparison of 27 hpf *rbm8a* to 21 hpf *magoh* mutant gene expression signatures (Fig 5A) is thus likely to compound the two factors described above resulting in smaller overlapping changes. It is also noteworthy that developing zebrafish embryos of the same genotype from the same clutch can show a 2-fold or more change in ~12% of genes [67], and embryos of the same genotype derived from different mothers show distinct mother-specific transcriptome signatures [67]. Such issues can further increase variation in gene expression estimates within and across biological replicates of the same genotype, amplifying differences among the two EJC mutants. Finally, some differences in gene expression profiles of *rbm8a* and *magoh* mutants could arise due to their EJC-independent functions (e.g. human RBM8A can bind to mRNA cap structure and may regulate decapping, [68,69]). In the future, the shared impact of the EJC core proteins in developing embryos can be better evaluated by comparing gene expression in specific cell types or tissues of zebrafish *rbm8a* and *magoh* mutants at the same developmental stage.

### The EJC is a critical component of NMD in zebrafish

Our finding that a significant fraction of genes upregulated in EJC mutant embryos are also upregulated in *upf1* knockdown embryos (Fig 5) suggests that EJC-dependent NMD is compromised in both *rbm8a* and *magoh* mutant embryos. The overlap between upregulated genes in EJC mutant and *upf1* morphants is small (Fig 5A and S3A Fig), likely due to differences in developmental timing or due to NMD-independent functions of Upf1 and EJC proteins. However, the genes within these overlaps include previously validated zebrafish NMD targets and orthologs of known NMD targets in mammals (Fig 5D and S2 Table). Furthermore, several known classes of NMD targets such as PTC-, uORF-, and 3′UI-containing transcripts are significantly upregulated in *rbm8a* and *magoh* mutant embryos (Fig 5E and 5F). A moderate enrichment of these classes of transcripts among the upregulated genes in EJC mutants (S3H and S3I Fig) suggests that many of the genes with these features are directly regulated by EJC-dependent NMD. Therefore, the EJC is important for the quality control function (i.e. suppression of aberrant PTC-containing transcripts) and the gene regulatory activity of the zebrafish NMD pathway, which further underscores the importance of EJC-dependent NMD for developmental and tissue-specific gene regulation [20,21,24,25]. An important future goal will be to expand on how EJC-dependent NMD regulates specific genes in particular cell types and tissues to control development.

### Proximal 3′UTR introns as a probable NMD-inducing feature

Several of our observations raise a possibility that proximal 3′UIs can induce NMD. Nearly 10% of all detectable zebrafish proximal 3′UI-containing genes, like distal 3′UI-containing genes, are ≥ 1.5 fold upregulated in EJC mutant and *upf1* morphant datasets (Fig 6B and 6C, S4D Fig), and a subset of these are upregulated in zebrafish embryos treated with the NMD inhibitor NMDI14 (Fig 6D). Further, a subset of proximal 3′UI-containing genes is also upregulated in mouse and human NMD- and EJC-compromised cells (Fig 7 and S5 Fig). Importantly, NMD inhibition specifically upregulates transcript isoforms that contain a proximal (or distal) 3′UI but not the 3′UI-lacking isoforms produced from the same genes (Fig 7F–7H). These observations are also consistent with previous reports of NMD susceptibility of a T cell receptor-*β* (*TCR-β*) reporter RNA where stop codon is only 10 nt upstream of the last exon-exon junction [70], and of triose phosphate isomerase reporter RNA with stop codon 40 nt upstream of the last exon-exon junction [71].

**Fig 9:**
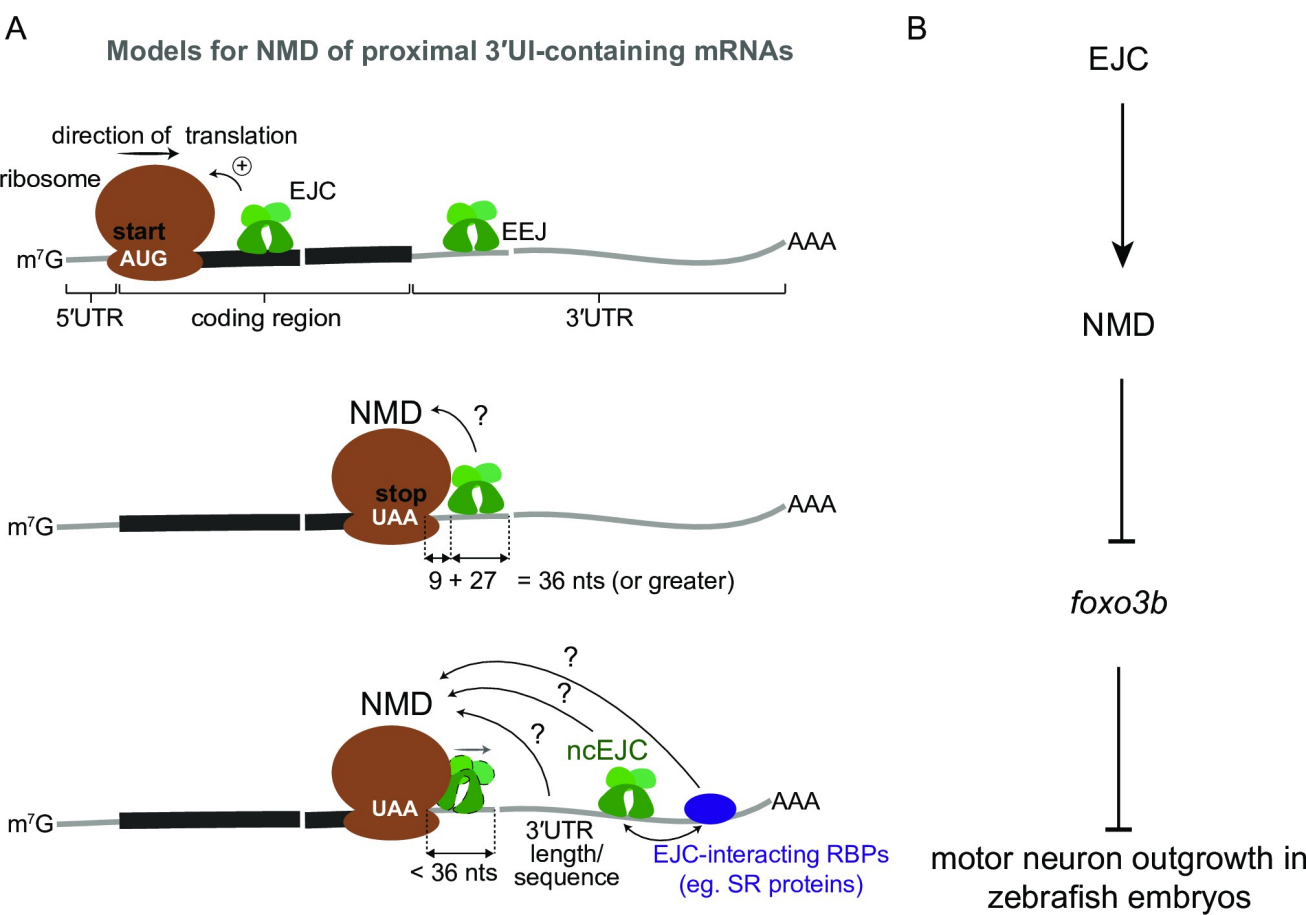
Possible models for EJC-dependent NMD of proximal 3′UI-containing transcripts and regulation of *foxo3b* function in zebrafish motor neurons. A. Top: EJCs can function to enhance NMD via translation stimulation. Ribosome (brown), direction of translation (straight arrow), start codon (‘AUG’ in white), EJC (green), exon-exon junction (EEJ), mRNA coding region (thick black line) and mRNA 5′ and 3′UTRs (thin gray lines) are labeled. Middle: a model for downstream EJC function in NMD of transcripts where the distance between stop codon (‘UAA’ in white) and downstream exon-exon junction is 36 nts or greater to accommodate both the EJC and the ribosome. Bottom: a model for NMD of transcripts where the distance between stop codon and downstream exon-exon junction is less than 36 nts. Displacement of stop codon-proximal EJC by the ribosome is shown. In this case other downstream factors such as a non-canonical EJC (ncEJC), EJC-interacting RBPs (e.g. SR proteins), or 3′UTR length and/or specific sequences may be responsible for NMD. B. A schematic depicting a model for EJC- and NMD-dependent regulation of *foxo3b* as a genetic pathway that is critical for zebrafish motor neuron development.

How can a proximal 3′UI lead to NMD? The prevalent 50-nt rule is presumed to account for the minimum distance required to accommodate a terminated ribosome at stop codon so that it does not interfere with the downstream EJC. Based on estimates that ribosome footprints at stop codons extend about 9 nts into the 3′UTR [e.g. see 58] and that the EJC 5′ boundary lies about 27 nts upstream of the exon-exon junction (Fig 1E), EJC deposited by a 3′UI located at least 36 nts downstream of a stop codon may not always be displaced by the terminating ribosome and could induce NMD via the currently accepted mechanism (Fig 9A). How introns within the first 35 nts of a 3′UTR might induce NMD is more perplexing. One possibility is that introns within 35 nts of the stop may trigger NMD via non-canonical EJCs present downstream in the 3′UTR [42,43] (Fig 9A). Additionally, EJC-interacting factors such as SR proteins deposited on 3′UTR sequences after 3′UI splicing may also recruit NMD-activating factors [42,43,72,73] (Fig 9A). Our data do not exclude additional possibilities for proximal 3′UIs to activate NMD in concert with other mechanisms that are intron-dependent (e.g. EJC-dependent translation enhancement or yet-unknown distal 3′UIs) or intron-independent (e.g. 3′UTR length or NMD-promoting sequences) (Fig 9A). Interestingly, in T cells in mice, PTC-containing *TCR-β* shows very strong intron-dependent NMD but is also downregulated >3-fold by an intron-independent mechanism [74]. Therefore, further studies are necessary to rigorously test if proximal 3′UIs, like distal 3′UIs, can act as an independent NMD-inducing feature.

We also observe that proximal 3′UI-containing genes are variably susceptible to reduced EJC/NMD function. For example, zebrafish *foxo3b* is significantly upregulated in EJC mutant and *upf1* morphants whereas human *FOXO3* is only mildly sensitive to reduced UPF1 levels in cell lines (Fig 6, S4 Fig, Fig 7 and S5 Fig) but shows robust upregulation upon EIF4A3 knockdown in HCT116 cells (Fig 7E). Furthermore, many vertebrate genes with proximal 3′UIs are not upregulated, or are even downregulated, upon diminished EJC/UPF1 function (Fig 6 and Fig 7). These observations, and previous findings of Wittkopp *et al* (2009) that a PTC introduced 6 nt upstream of the last exon-exon junction of a reporter RNA does not elicit NMD in zebrafish cultured cells [54], show that many proximal 3′UI-containing transcripts may not be subject to NMD at all. Interestingly, similar to proximal 3′UI-containing genes, distal 3′UI-containing genes also show a variable susceptibility to EJC/NMD-deficiency (Fig 6B and 6C, Fig 7C). The variability and/or non-responsiveness of some 3′UI-containing genes to EJC/NMD manipulations could be due to their low expression at developmental stage/cell type investigated or due to their variable sensitivity to EJC/NMD protein levels. Further, downregulation of proximal 3′UI-containing genes could result from indirect effects of compromised EJC/NMD function. It is also possible that some 3′UI-containing genes actively evade NMD via 3′UTR-bound proteins [75]. Thus, regulation of mRNA stability by 3′UIs is likely to be a net outcome of a combinatorial control of NMD by multiple determinants of 3′UTR RNP composition, a model that requires further validation on gene-by-gene basis.

### EJC-dependent NMD of *foxo3b* is critical for zebrafish motor axon outgrowth

Certain genes maintain proximal 3′UIs across vertebrate evolution (Fig 7 and Fig 8) despite much faster rates of intron loss from 3′UTRs compared to coding region [76,77], suggesting that proximal 3′UIs may play an important role in gene regulation and cellular function. *foxo3b* is one such example that is regulated via EJC-dependent NMD in zebrafish embryos (Fig 6) and cultured human cells (Fig 7), and such control is critical for motor axon outgrowth (Fig 8 and Fig 9B). Therefore, of the hundreds of NMD targets identified in multiple cell types and organisms, *foxo3b* is among the handful of genes such as *Robo3*.*2* in mouse commissural axons [20] and *Smad7* in cultured embryonic stem cells [62], whose regulation by NMD impacts cell fate and differentiation *in vivo*. *foxo3b* encodes a forkhead box transcription factor that acts as a hub for integration of several stress stimuli, and functions in processes such as cell cycle, apoptosis, and autophagy [78,79]. In zebrafish, Foxo3b has been implicated in survival under hypoxic stress [66], inhibition of antiviral responses [65], and canonical *wnt* signaling inhibition [80]. FOXO3, the mammalian ortholog of Foxo3b, physically interacts with p53, and both act synergistically to induce apoptosis in response to stress [81]. In addition, several pro-apoptotic genes (e.g. *bim*, *bbc3*, *gadd45a*) are direct FOXO3 transcriptional targets (S6C Fig) [82,83]. Thus, the regulation of *foxo3b* by EJC-dependent NMD (Fig 6 and Fig 8) can directly impact cell survival. The elevated levels of Foxo3b in EJC mutant embryos may cause motor axon growth defects due to reduced Wnt signaling [80,84], increased neural cell death, and/or other cell-autonomous or non-cell-autonomous reasons. Following the loss of *foxo3b* function in EJC mutant embryos, the partial reversal of motor axon length (Fig 8) parallels the rescue of neural apoptosis in mouse EJC mutant embryos upon brain-specific *p53* ablation [30,31], and the reversal of cell death in NMD-defective flies and human cell lines upon reduced activity of *GADD45A* [85]. Therefore, *foxo3b* is a critical NMD target in zebrafish motor neurons, and may serve as an example for how 3′UIs can modulate 3′UTR RNP composition, mRNA stability and protein production during development (Fig 9).

## Materials and methods

### Ethics statement

Animal experiments were performed in accordance with institutional and national guidelines and regulations and were approved by the Ohio State University Animal Care and Use Committee (Protocol number: 2012A00000113-R2).

### Animal stocks, lines, and husbandry

Adult zebrafish (*Danio rerio*) were housed at 28.5°C on a 14 hour light/10 hour dark cycle and embryos were obtained by natural spawning or *in vitro* fertilization. Embryos were raised at both 25°C and 28.5°C and were staged according to Kimmel *et al*. (1995) [87]. *rbm8a*^oz36^ and *magoh*^oz37^ lines generated using CRISPR/Cas9 mutagenesis (described below) in the AB strain. The *foxo3b*^ihb404^ line [65] was obtained from the Xiao lab at the Chinese Academy of Sciences, Wuhan, China.

### CRISPR/Cas9 mutagenesis

An optimal CRISPR target site in the coding sequence of *rbm8a* and *magoh* was identified using the ZiFit Targeter software package [88,89]. gRNAs were designed and synthesized as described [47]. *rbm8a-* or *magoh*-targeting gRNA was co-injected with *Cas9* mRNA [90] into 1-cell stage embryos (60 pg gRNA and 160 pg *Cas9* mRNA).

*rbm8a* gRNA target site: (5′-GGGAGGCGAAGACTTTCCTA-3′)

*magoh* gRNA target site: (5′-GGTACTATGTGGGGCATAA-3′)

Injected embryos were raised to 24 hpf at which time embryos were individually screened by high-resolution melting analysis (HRMA) to assess target site mutation efficiency in somatic cells. Remaining embryos were raised and crossed to AB wild-type adults; F1 adults were screened for germline transmission of CRISPR-induced mutations using HRMA. HRMA revealed unique *rbm8a* and *magoh* mutant alleles transmitted by multiple F0 founders. We recovered the *rbm8a*^*oz36*^ *and magoh*^*oz37*^ alleles and outcrossed the heterozygotes to the AB wild-type strain for two generations before intercrossing for phenotypic analyses. Sequences of primers used for genotyping are listed in S4 Table.

### EJC mutant and *foxo3b*^*ihb404*^ mutant embryo and adult genotyping strategy

Individual embryos and adult fin tissue were lysed in 50 μl 1M NaOH for 15 mins at 95°C followed by incubation on ice for 5 minutes at 4°C, and then neutralized with 5 μl of 1M Tris-HCl pH 8. For genotyping fixed embryos, heads were removed into ThermoPol buffer (20 μl) and treated with 2 mg/ml ProK at 55°C for 3 hours to extract DNA. 1 μl of DNA extract was used as a template in a 20 μl PCR with Taq polymerase according to the manufacturer's protocol (NEB). For genotyping *rbm8a*^*oz36*^ *and foxo3b*^*ihb404*^ mutant embryos, PCR products were digested with 20 units of XmnI and XcmI respectively (NEB) to distinguish cleavable mutant from un-cleavable wild-type amplicons. Digested products were analyzed on a 1% agarose gel stained with Gel Red (Biotium). For genotyping *magoh*^*oz37*^ mutant embryos, PCR products were analyzed by separation of mutant and wild-type alleles on a 2% agarose gel stained with Gel Red (Biotium). Primer sequences are listed in S4 Table.

### Acridine orange staining and immunohistochemistry

Embryos were incubated in 1:5000 acridine orange solution for 1 hr at 28.5°C (stock: 6 mg/ml, Sigma-Aldrich) followed by 2X washes in fish system water. For immunohistochemistry, embryos were processed following standard protocols using 4% PFA fixation, permeabilization using acetone, and incubation in blocking solution for 1 hour. EJC mutant embryos and wild-type siblings at 24 hpf and 26 hpf were incubated in 2% BSA/2% goat serum/1% DMSO/0.1% Tween-20/PBS blocking solution with 1:100 dilution anti-SV2 (DSHB) and 1:1000 anti-A4.1025 (DSHB) primary antibodies and AlexaFlour (Molecular Probes) secondary antibodies. Embryos were stained with Alexa Fluor 488-conjugated α-Bungarotoxin (Thermo Fisher) incubation in a 1:200 blocking solution post primary and secondary antibody staining. All images were centered on the region above the end of the yolk tube which included somites 12–16 at 24 hpf and somites 16–20 at 26 hpf.

### Microscopy and Imaging

Immuno-stained embryos were dissected and mounted in Fluoromount-G (SouthernBiotech) and imaged at 40X magnification using MetaMorph software (Molecular Devices) on an Andor SpinningDisc Confocal Microscope (Oxford Instruments) with Nikon Neo camera. Live images of EJC mutant and wild-type sibling embryos were taken by mounting embryos in 3% methylcellulose and imaging with a AxioCam camera on a Zeiss upright AxioPlan2 microscope.

### Zebrafish NMDI14 inhibitor treatment

NMDI14 (Sigma) stock solution was made in DMSO as per manufacturer’s instructions. AB wild-type zebrafish embryos were dechorionated on agarose-coated 10 cm plates. At 3 hpf, NMDI14 was added to a final concentration of 4.8 μM. At 24 hpf, embryos (20/treatment) were rinsed with fresh fish water and added to 500 μl of Trizol (Thermo Fisher Scientific) for RNA preparation.

### Immunoblot analysis

SDS-PAGE gels and western blots were performed using the standard mini-PROTEAN tetra system (Bio-Rad). All western blots were stained using infrared fluorophore-conjugated secondary antibodies and were scanned on a LI-COR Odyssey CLx imager. Protein quantification was performed using Image Studio software (v5.2.5).

### Quantification of paralysis and motor axon length

At 24 hpf, EJC mutant and wild-type sibling embryo movements were scored under the dissecting microscope by counting the number of tail contractions per minute. For motor neuron axon quantification, immunofluorescence images of 26 hpf EJC mutant and wild-type sibling embryos were stained with anti-SV2 as described above. Images were imported into Fiji (ImageJ v2) and motor axon length was quantified using the Simple Neurite Tracer plugin [91].

### RNA-Immunoprecipitation-Seq and RNA-Seq sample collection

At 24 hpf, zebrafish embryos (n = 800 embryos/IP) were triturated using a 200 μl pipette and washed to remove yolks as previously described [92], followed by flash freezing the tissue in liquid nitrogen. Whole embryo tissue was lysed and sonicated in 800 μl of hypotonic lysis buffer (HLB) [20 mM Tris-HCl pH 7.5, 15 mM NaCl, 10 mM EDTA, 0.5% NP-40, 0.1% Triton X-100, 1 mM Aprotinin, 1 mM Leupeptin, 1 mM Pepstatin, 1 mM PMSF]. Lysates were sonicated using a microtip for 7 seconds, NaCl was increased to 150 mM, and RNase I was added to 100 μg/ml. Following a 5-minute incubation on ice, cell lysates were cleared by centrifugation at 15,000 × g. The sample was split into 2 tubes (400 ul each) and the volume was increased to 2 mL by addition of isotonic lysis buffer. Complexes were captured on Protein G Dynabeads (Thermo Fisher) conjugated to IgG or α-Rbm8a for 2 hours at 4°C. Complexes were washed in isotonic wash buffer (IsoWB) [20 mM Tris-HCl pH 7.5, 150 mM NaCl, 0.1% NP-40] and eluted in clear sample buffer [100 mM Tris-HCl pH 6.8, 4% SDS, 10 mM EDTA, 100 mM DTT]. The proteins were eluted in 20 μl of clear sample buffer [100 mM Tris-Hcl pH 6.8, 4% SDS, 10mM EDTA, 100 mM DTT] and 10 μl of the sample was used to separate the proteins via SDS-PAGE and analyze by western blotting. The remaining 10 μl of the sample was used for RNA extraction using Phenol-Chloroform-Isoamyl alcohol precipitation. RNA was resuspended in 10 μl of RNase-free water. 1 μl of the RNA was end-labeled with γ-^32^P-ATP and then run on a denaturing 20% urea PAGE gel to assess quality while the remainder was used for RNA-Seq library preparation.

For RNA-Seq sample collection, EJC mutant and wild-type sibling embryos (N = 25) were harvested at 21 and/or 27 hpf and lysed in 500 μl Trizol (Thermo Fisher Scientific). Heterozygote and wild-type embryos (wild type sibling embryos) were mixed together for the control RNA-Seq dataset because heterozygotes are morphologically indistinguishable from wild-type embryos, they can only be distinguished by the DNA genotyping strategies described above. RNA was extracted following manufacturer standard procedures.

### RIP-Seq and RNA-Seq library preparation

For RIP-Seq, RNA extracted from ~90% of RIP eluate was used to generate strand-specific libraries. For RNA-Seq libraries, 5 μg of total cellular RNA was depleted of ribosomal RNA (RiboZero kit, Illumina), and subjected to base hydrolysis. RNA fragments were then used to generate strand-specific libraries using a custom library preparation method [93]. Briefly, a pre-adenylated miR-Cat33 DNA adapter was ligated to RNA 3′-ends and used as a primer binding site for reverse-transcription (RT) using a special RT primer. This RT primer contains two sequences linked via a flexible PEG spacer. The DNA with a free 3′-end contains sequence complementary to a DNA adapter as well as Illumina PE 2.0 primers. The DNA with a free 5′-end contains Illumina PE 1.0 primer sequences followed by a random pentamer, a 5 nt barcode sequence, and ends in GG at the 5′-end. Following RT, the extended RT primer was gel purified, circularized using CircLigase (Illumina), and used for PCR amplification using Illumina PE 1.0 and PE 2.0 primers. All DNA libraries were quantified using an Agilent Bioanalyzer to determine DNA length and a Qubit Fluorometer to quantify DNA amount. Libraries were sequenced on an Illumina HiSeq 2500 platform in the single-end format (50 nt read lengths). For each RNA-Seq experiment consisting of an EJC mutant and its WT sibling at a given time-point, three biological replicates were sequenced per genotype.

### Zebrafish EJC mutant embryo RIP-seq and RNA-Seq data analysis

#### Adapter trimming and PCR duplicate removal

After demultiplexing, fastq files containing unmapped reads were first trimmed using Cutadapt (v2.3). A 12 nt sequence on read 5′-end consisting of a 5 nt random barcode sequence, 5 nt identifying barcode, and a CC was removed. The random barcode sequence associated with each read was saved for identifying PCR duplicates down the line. Next, as much of the 3′-adapter (miR-Cat22) sequence TGGAATTCTCGGGTGCCAAGG was removed from the 3′-end as possible. Any reads less than 20 nts in length after trimming were discarded.

#### Alignment and removal of multi-mapping reads

For RIP-Seq, following adapter trimming, reads were aligned with HISAT2 v2.1.0 (Kim *et al*., 2015) using 24 threads to zebrafish GRCz10. After alignment, reads with a HISAT2 mapping score less than 60 were removed, i.e. all multi-mapped reads were discarded. Finally, all reads mapping to identical regions were compared for their random barcode sequence; if the random sequences matched, such reads were inferred as PCR duplicates and only one such read was kept.

For RNA-Seq, adapter-trimmed libraries were aligned to the zebrafish genome using TopHat2 [94] (v2.0.14 and default options:—read-mismatches 2,—red-gap-length 2,—read-edit-dist 2,—min-anchor-length 8,—splice-mismatches 0,—num-threads 2 (not default),—max-multihits 20) and the GRCz10 genome assembly. Read count followed by differential expression analysis was conducted as stated in the Love *et al*. 2018 RNA-Seq workflow.

#### RIP-Seq data downstream analyses

First, by comparing aligned reads to a GRCz10 exon annotation obtained through Ensembl BioMart we determined the 5′ and 3′ end distribution of RIP-Seq reads and the meta-exon distributions of RIP-Seq reads. The primary reference transcriptome was obtained from Ensembl BioMart. For each gene, the principal transcript isoform (transcript with an APPRIS P1 annotation) was selected for all analyses concerning the specificity of the RIP-Seq replicates. These analyses include calculation of RPKMs for the major APPRIS P1 isoform and comparison of intronic RPKMs to exonic RPKMs as well as intron-less transcript RPKMs to multi-exon transcript RPKMs.

#### RNA-Seq differential expression analysis

Differential expression analysis using EJC mutant embryos and wild-type RNA-Seq data was conducted based on the RNA-Seq workflow published by Love *et al*. 2018 [95].

First, to create count-matrices for each RNA-Seq experiment, the GenomicAlignments and SummarizedExperiment software [96,97] were used to count reads mapping per gene for each RNA-Seq bio-replicate. The count matrix was filtered to remove all genes with zero counts in all samples before differential expression (DE) analysis using DESeq2 [98]. At least one, if not all of the biological replicates for each RNA-Seq experiment were sequenced during a separate deep-sequencing run. These differences in sequencing runs introduced some variability among the replicates. To account for the variability among our RNA-Seq bio-replicates during differential expression analysis we used the RUV-seq R package [99]. Usual methods of normalization only account for sequencing depth but RUV-seq methods can be used to normalize libraries for library preparation and other technical effects. We used the RUVs method of the RUV-seq package which utilizes the centered counts (the counts of genes unaffected by our covariates of interest such as the sample genotype) to determine a normalization factor for each library. The count matrix was imported into DESeq2, and the RUVs normalization factors and genotype were used in the design formula to construct the DESeq dataset for gene-level differential expression analysis. We used the LRT test with all default DESeq2 settings to identify genes differentially expressed between mutant and wild-type samples. To correct for multiple testing in the DE analysis we used Benjamini-Hochberg (BH) adjustment with independentFiltering set to false. We decided to set independentFiltering to false because we are interested in studying NMD targets which are most likely to have low read counts in wild-type embryos. In the case of the *rbm8a*^-/-^ 21 hpf and 27 hpf datasets the histogram of all p-values showed a hill-shaped distribution. In order to account for this distribution, as per the suggestion made in RNA-Seq workflow [95], we used fdrtool [100] for multiple testing and determined adjusted p-values using default fdrtool settings.

### Gene Ontology enrichment analysis

The PANTHER14.0 [86] tool was used to identify significantly enriched biological process GO terms in genes that are found to be significantly differentially expressed in EJC mutant embryos by DESeq2. The PANTHER tool was also used to identify significantly enriched biological process GO terms in proximal 3′UI genes in zebrafish and humans. For all analyses the PANTHER’s Benjamini-Hochberg correction was used to calculate adjusted p-values.

### Overlap analysis

The universal and test sets were chosen to be the set of Ensembl gene IDs which were assigned an adjusted p-value post-DESeq2 analysis. After determining a universal dataset for each RNA-seq dataset, the smallest universal set for the comparison in question was chosen. The significance of overlap was calculated using a hypergeometric test using the R statistical software.

### STRING network analysis

The STRING database [101] was used to identify connections between proteins encoded by proximal 3′UI genes with default high confidence settings (minimum required interaction score = 0.7). The clusters shown were created after the Markov Cluster Algorithm (MCL) inflation parameter was set to 3 clusters.

### Human and mouse RNA-Seq data analysis

SRA files were downloaded from sources specified in [62,63]. Fastq files generated from the SRA files were mapped using TopHat2 (version 2.1.1) using the same settings described above for zebrafish alignment. Count matrices were generated using the GenomicAlignments and SummarizedExperiment packages. The count matrices were imported into DESeq2 for differential expression analysis using the LRT test, BH adjustment and with independentFiltering set to false.

### Identification of uORF genes in zebrafish, human and mouse

We selected for uORFs which were categorized as “functional uORFs” in Johnstone *et al*. 2016 [59] based on RNA-Seq and ribosome profiling. The following filters were applied to select for uORFs that show evidence of translation at 24 hpf: 5′UTR RPF RPKM ≥ 5, RNA-Seq RPKM≥ 5, RPF RPKM ≥ 5, ORF translation efficiency at 24 hpf >1.

### Identification of 3′UTR intron containing genes in zebrafish, mouse, and human

A table describing exon starts, exon ends, CDS start, CDS end, strand and APPRIS annotation was downloaded from the Ensembl database for all transcripts in zebrafish (GRCz10), human (GRCh38) and mouse (GRCm38). All transcripts with any level of APPRIS annotation [56] were included. We then identified transcripts that contain introns in the 3′UTRs by subtracting exon start coordinates from the CDS end coordinates in a strand specific manner. We then determined the distance of the farthest 3′UTR intron to the stop codon; based on the distance (< or ≥ 50 nts) as well as the number of 3′UTR introns the transcripts were classified into proximal and distal categories. Proximal transcripts were defined by the presence of only one 3′UTR intron which is within 50 nts of the normal stop codon. Distal transcripts were defined by the presence of one 3′UTR intron which is more than 50 nts away from the stop codon or by the presence of more than one 3′UTR intron irrespective of the distance of the nearest 3′UTR intron to the stop (i.e.If a gene encodes transcript with both proximal and distal 3′UIs, it is classified in the latter group.). For all fold-change analyses, we defined distal/proximal 3′UI-containing genes as those that encode one or more distal/proximal 3′UI+ transcripts. The distal 3′UI-containing genes that did not have an APPRIS annotation but were annotated with an ‘NMD biotype’ in the Ensembl database were included as a separate group in the analyses included in Fig 5 and the group was named as NMD biotype.

### Analysis of NMD-sensitive and NMD-insensitive isoforms of 3UI-containing genes

The transcript level quantification data were obtained from S4 Table of Baird *et al*. (57). After removing transcripts that lack q-values in *UPF1* knockdown compared to the control, transcripts were separated based on the presence or absence of a PTC as indicated in the table. Among the PTC-containing transcripts, those that contain a 3′UI and are the only detectable 3′UI-containing transcript from the corresponding gene were selected as distal 3′UI-containing isoforms based on our classification described above. Among the PTC-lacking transcripts, those that contain a proximal 3′UI and are the only detectable 3′UI-containing transcript of the corresponding gene were selected as proximal 3′UI-containing isoforms based on our classification. Finally, we selected the most abundant (highest TPM value in the control dataset) 3′UI-lacking isoform of the selected proximal and distal 3′UI-containing genes as control groups for analysis in Fig 7F and 7G.

### Mammalian cell culture knockdown experiments

HCT116 cells were seeded into 12 well plates (10^5^ cells/ well) in McCoy’s 5A media and 15 pmol siRNA was reverse transfected using 1.6 μl lipofectamine RNAiMAX reagent (Thermo Fisher Scientific) per well. Knockdown was carried out for 48 hours with a media change after 24 hours. Cells were harvested in hypotonic lysis buffer (described above), 30% of the cell lysate was saved to check the efficiency of knockdown (S5J Fig) while the rest was added to TRI Reagent (Sigma) for RNA extraction. The siRNAs used in this study are listed below:

Hs_*RBM8A*_5 FlexiTube siRNA, no modification, 20 nmole (SI03046533, Qiagen)

All Stars Negative Control siRNA, no modification, 20 nmole (SI03650318, Qiagen)

*UPF1*_1879: AAG AUG CAG UUC CGC UCC AUU

*EIF4A3*_187: CGA GCA AUC AAG CAG AUC AUU

### Zebrafish *upf1* knockdown experiment and RNA-Seq

For conducting *upf1* knockdown in zebrafish, 2 ng of a splice blocking morpholino (MO) diluted in 0.2 M KCl with 0.1% phenol red was injected into 1-cell stage embryos. The *upf1* MO used was previously published and named *upf1* MO2 [54] with a sequence of 5′-TTTTGGGAGTTTATACCTGGTTGTC-3’. Morpholino was synthesized by Gene Tools, LLC. Uninjected wild-type control embryos (n = 30) and injected morphants (n = 30) were raised for 12 hours at 28.5°C and lysed in 500 μl Trizol following manufacturer’s procedures (Thermo Fisher Scientific). After RNA-extraction, double-stranded cDNA was synthesized following Illumina's TruSeq protocol per manufacturer’s instructions. Briefly, mRNA was purified from 1 μg total RNA using Dynabeads oligo(dT)_25_ magnetic beads (Thermo Fisher Scientific) followed by clean-up with AMPure XP SPRI beads (Beckman Coulter). mRNA was then fragmented for 5 minutes at 70°C using Ambion's RNA Fragmentation Reagent (AM8740) followed by an additional clean-up step using AMPure XP SPRI beads. Fragmented RNA was reverse transcribed using random primers and SuperScript III (Thermo Fisher Scientific). After second strand synthesis, end repair, and 3' end adenylation per the TruSeq protocol (Illumina, Inc.), libraries were constructed using an Apollo 324 automated library system. After Illumina adapter ligation and amplification, all DNA libraries were quantified using an Agilent Bioanalyzer instrument to determine DNA length and a Qubit Fluorometer to quantify DNA amount. 10 cycles of amplification was performed prior to sequencing on an Illumina HiSeq 2000 system in the paired-end format (100 nt read lengths). All experiments were performed in biological duplicate.

#### RNA-seq data analysis

Trimmed reads were obtained from the sequencing core and then mapped to the GRCz10 genome assembly using TopHat2 as described above. Differential gene expression analysis was also performed as described above using DESeq2. RUV-Seq and fdrtool corrections were not required.

### Quantitative RT-PCR

Zebrafish embryos and mammalian cells were harvested in Trizol. RNA was isolated using standard Trizol procedures, followed by DNase treatment, purification with Phenol:Chloroform: Isoamyl alcohol (25:24:1, pH 4.5) and resuspension in RNase-free water. 1.5 μg of RNA was reverse transcribed using oligo-dT and Superscript III (Invitrogen). After reverse transcription of RNA, the samples were treated with RNase H (Promega) for 30 min at 37°C. For each qRT-PCR 30 ng of cDNA was mixed with 5 μl of 2X SYBR Green Master Mix (ABS), 0.2 μl of a 10 mM forward and reverse primer each (defrosted once) in a 10 μl reaction. The qRT-PCRs were performed in triplicate (technical) using primers described in S4 Table. Reference genes for relative quantification were *mob4* in zebrafish [102] and TATA-binding protein (*TBP*) in human cells. Fold-change calculations were performed by the ΔΔC_t_ method. Fold-changes from three biological replicates were used to determine the standard error of means. The p-values were calculated using Welch t-test in the R statistical computing software.

### Quantification and statistical analysis

All western blots were performed using infrared fluorophore conjugated secondary antibodies and were scanned on a LI-COR Odyssey CLx imager. Protein quantification was performed using Image Studio software (v5.2.5). Northern blot autoradiograms were scanned using Fuji FLA imager and quantified using ImageQuant TL software (v7.0). Average and standard error of means in the observed signal was determined for data from at least three biological replicates.

## Supporting information

S1 Fig Related to Fig 1. A. Multiple sequence alignments of Eif4a3, Rbm8a and Magoh protein sequences from organisms on the left. Consensus sequence is at the bottom with upper case letters indicating identity and lower case letters indicating similarity. Green indicates complete identity across all species, yellow and blue indicate the identical and unique amino acids in the regions with similarity. Identity between human and zebrafish EJC proteins: Eif4a3 (97%), Rbm8a (93%) and Magoh (100%). B. Western blot detecting proteins listed on the left in RNase I-treated zebrafish embryo total extract (TE, lane 1), depleted extract (DE, lanes 2, 4 and 6) and immunoprecipitates (IP, lanes 3, 5 and 7) with the Rbm8a antibody. Detergents supplemented to increase IP stringency are indicated on top of each lane. Optimized IP condition used in S1C is indicated by the dashed red box. C. Autoradiogram of γ^32^P 5′-end labeled RNAs from anti-Rbm8a RIP elution (lane 4) as well as indicated size-markers which include the low-molecular weight single-stranded DNA ladder (lane 1), 0.1 pmol 28 nt synthetic RNA (lane 2) and 100 bp DNA ladder (lane 3). D. Scatter plots comparing read counts for each gene in a pair of RIP-Seq replicates. The replicates (Rep1, Rep2, and Rep3) are indicated on the x- and y-axes. A pseudocount of 0.0001 was added to all genic read counts before log_2_ transformation. Pearson correlation coefficient (r) and p-value for the correlation test for each comparison is on the top left of each plot. E. Genome browser screenshots showing read coverage of Rbm8a RIP-Seq (only Rep 3, the deepest replicate is shown) in green and RNA-Seq in gray of select highly-expressed genes, *krt4*, *eef2b*, *eif4g1a*, and *hist1h4l (intron-less gene)*.
(TIF)

S2 Fig Related to Fig 2. A. Whole mount images of live 19 hpf EJC mutant embryos and WT sibling embryos stained with acridine orange. B. Whole mount images of live EJC mutant embryos and WT siblings at 21 hpf. C. Whole mount images of live EJC mutant embryos and WT siblings at 27 hpf. D. Whole mount images of live EJC mutant embryos and WT siblings at 32 hpf. Head necrosis is indicated by the dashed circle.
(TIF)

S3 Fig Related to Fig 5. A. Venn diagram showing the overlap of genes that are significantly upregulated in EJC mutant embryos and *upf1* morphant embryos at 24 hpf [55]. Hypergeometric test p-values for each comparison are also shown. B. MA plot showing the genes that are altered in expression (fold change > 1.5 and FDR < 0.05 in red and unchanged genes in gray) in *upf1* morphant embryos compared to control embryos at 12 hpf. The number of significantly upregulated genes is at the top right and the number of downregulated genes is at the bottom right. C. Venn diagram showing the overlap of significantly upregulated genes in *upf1* morphant embryos at 24 hpf [55] and *upf1* morphant embryos at 12 hpf. Hypergeometric test p-value for the comparison is indicated. D. Empirical CDF plot showing the fold changes in *upf1* morphant embryos (24 hpf) [55] of upregulated (blue) and unchanged genes (black) in 21 hpf *magoh* mutant embryos. Kolmogorov-Smirnov (KS) test p-value for differences in the two distributions are indicated at the bottom of the class descriptions. E. Empirical CDF plot as in S3D for upregulated (red) and unchanged genes (black) in *rbm8a* mutant embryos at 21 hpf. F. Empirical CDF plot as in S3D for upregulated (red) and unchanged genes (black) in *rbm8a* mutant embryos at 27 hpf. G. Empirical CDF plot showing the fold changes in *upf1* morphants (12 hpf) of upregulated (red) and unchanged genes (black) in *rbm8a* mutant embryos at 21 hpf. H and I. Proportion of uORF-containing (H) or 3′UI-containing (I) genes in the total number of genes showing significant (FDR < 0.05) fold changes in *rbm8a* mutant, *magoh* mutant and *upf1* morphant embryos. Genes are divided into four categories based on their log_2_ fold change: >1.5, 1.5 to 0, 0 to -1.5 and < 1.5.
(TIF)

S4 Fig Related to Fig 6. A. Histogram depicting the frequency of all zebrafish 3′UI transcripts in Ensembl GRCz10 (with APPRIS annotation) as a measure of the distance of the 3′UI from the stop codon. Data are shown in 5 nts bins and bins beyond 500 nts are not shown. Bins of proximal 3′UI genes are in blue and distal 3′UI bins are in gray. Inset: Histogram of all zebrafish proximal 3′UI transcripts binned by 1 nt. B. PANTHER14.0 [87] gene ontology (GO) term enrichment analysis of proximal 3′UI-containing genes (top, shades of blue) and all 3′UI-containing genes (bottom, shades of gray). All significant terms (Benjamini-Hochberg corrected p-value < 0.05) are shown for each set. C. A scatter plot showing gene-level fold change (FC) for transcripts with proximal 3′UI (dark blue: FC > 1.5 and light blue: FC < 1.5) and distal 3′UI (black: FC > 1.5 and gray: FC < 1.5) in *rbm8a* mutant embryos at 21 hpf compared to wild-type siblings. Genes encircled in red also contain a uORF as determined from a previously published dataset (see Materials and Methods). D. A scatter plot as in C showing fold changes of 3′UI-containing genes for 12 hpf *upf1* morphants compared to wild-type control embryos. E. Integrated genome browser (IGV) screenshots of Sashimi plots showing RNA-seq reads observed for *foxo3b*, *phlda2* and *cdkn1ba* in four zebrafish 24 hpf whole embryo RNA-seq datasets (as labeled on figure in different colors) obtained from the DanioCode consortium. Range of the number of reads mapping to the genes are indicated to the left of each track in black. Number of junction reads are indicated at the spliced junction in the color corresponding to the specific track. In case of *foxo3b*, due to the length of the second intron the screenshots of the first two and the last two exons are shown separately. F. Empirical CDF plot showing the fold changes in *magoh* mutants (21 hpf) of genes upregulated that contain a proximal (green) or distal (light purple) 3′UI or intron-less genes (black). Genes that also contained an uORF were excluded from analysis. Kolmogorov-Smirnov (KS) test p-value for differences in fold changes between the two groups is indicated on the bottom right. G. CDF plot as in F showing the fold changes in *rbm8a* mutants (27 hpf) of genes upregulated that contain a proximal (green) or distal (light purple) 3′UI or intron-less genes (black). Kolmogorov-Smirnov (KS) test p-value for differences in fold changes between the two groups is indicated on the bottom right.
(TIF)

S5 Fig Related to Fig 7. A. Histogram showing the frequency of all APPRIS 3′UI-containing transcripts in human GRCh38 as a measure of the distance of the 3′UI from the stop codon. Data are grouped in 5 nt bins from 1–500 nts. Proximal 3′UI-containing gene bins are indicated in blue; distal 3′UI-containing gene bins are indicated in gray. Red dotted line indicates distance from stop codon to farthest 3′UI = 50 nts. Histogram of transcripts from Ensembl annotation is shown in C. B. Histogram as in A of mouse proximal and distal 3′UI-containing transcripts in mouse GRCm38. Histogram of transcripts from Ensembl annotation is shown in D. C. Histogram as in A of proximal and distal 3′UI-containing human transcripts from Ensembl annotation of GRCh38. D. Histogram as in A of proximal and distal 3′UI-containing mouse transcripts from Ensembl annotation of GRCm38. E. PANTHER14.0 [86] gene ontology (GO) term enrichment analysis of human APPRIS proximal 3′UI-containing genes (shades of blue) and all human APPRIS 3′UI-containing genes (shades of gray). All significant terms (Benjamini-Hochberg corrected p-value < 0.05) are shown for each set. F. GO term enrichment analysis as in E of mouse APPRIS 3′UI-containing genes. G. A scatter plot showing gene-level fold changes for all Ensembl-annotated transcripts with proximal 3′UI (dark blue: FC > 1.5 and light blue: FC < 1.5) and distal 3′UI (black: FC > 1.5 and gray: FC < 1.5) in *UPF1* knockdown human embryonic kidney cells (HEK293) compared to control cells using previously published data [57]. Genes encircled in red also contain a uORF as determined from a previously published dataset (see Methods). H. A scatter plot showing gene-level fold changes for all APPRIS-annotated transcripts with proximal 3′UI (dark blue: FC > 1.5 and light blue: FC < 1.5) and distal 3′UI (black: FC > 1.5 and gray: FC < 1.5) in *UPF1* knockdown human embryonic stem cells (hESCs) compared to control cells using previously published data [62]. Genes encircled on red also contain a uORF as determined from a previously published dataset (see Materials and Methods). I. Scatter plot as in E showing gene-level fold changes of APPRIS-annotated mouse 3′UI-containing transcripts in *Smg6*^-/-^ knockout mouse embryonic stem cells (mESCs) compared to wild-type cells [63]. J. Western blots showing representative protein knockdown in one replicate for Fig 7E.
(TIF)

S6 Fig Related to Fig 8. A. Semi-quantitative RT-PCR shows transcript levels of *foxo3b*, *eif4a2* and *rpl13* (loading control) in *rbm8a* mutant and wild-type sibling embryos at 21 and 27 hpf. B. Western blots (on the left) show levels of Foxo3b, Rbm8a, Magoh, and Tubulin in *rbm8a* mutant embryos (lane 2) compared to WT siblings (lane 1) at 27 hpf (N = 20 embryos per genotype). Right: dot plot showing Foxo3b levels normalized to tubulin levels in *rbm8a* mutant embryos and WT siblings at 27 hpf in three biological replicates. Error bars: standard error of means. Welch’s t-test p-values are indicated at the top. C. Bar graph showing log_2_ fold changes of known Foxo3b transcriptional targets that show a significant upregulation (FDR < 0.05) in EJC mutant RNA-Seq datasets. Foxo3b targets are from Morris *et al*. 2015 [64]. A pound symbol indicates statistically non-significant log_2_ fold change with FDR > 0.05. D-G. Confocal images showing Myh1 immunofluorescence using anti-A4.1025 in somites 12–16 of WT sibling (D), *rbm8a*^-/-^ mutant (E), *rbm8a*^-/-^; *foxo3b*^+/-^ mutant (F), and *rbm8a*^-/-^; *foxo3b*^-/-^ mutant (G) embryos. (N = 5 embryos/genotype). H-K. Merged confocal images showing motor neurons (red; detected by anti-SV2 staining) and acetylcholine receptors (green; detected by alpha-bungarotoxin staining) in somites 12–16 of WT sibling (H), *rbm8a*^-/-^ mutant (I), *rbm8a*^-/-^; *foxo3b*^+/-^ mutant (J), and *rbm8a*^-/-^; *foxo3b*^-/-^ mutant (K) embryos. Neuromuscular junctions in the merged image are yellow. White arrowheads point to the distal end of the motor neuron. (N = 5 embryos/genotype). Scalebar in K (for panels D-K) is 100 nm. L. Boxplots showing quantification of motor axon length in embryos of genotypes indicated along the x-axis) (4 motor neurons/embryo and 5 embryos/genotype). Welch’s t-test p-values are indicated at the top.
(TIF)

S1 Table RIP-Seq and RNA-Seq data summary. (XLSX)

S2 Table Genes upregulated in RNA-Seq datasets represented in Fig 5A. (XLSX)

S3 Table Master list of zebrafish, mouse and human 3′UI-containing genes. (XLSX)

S4 Table List of oligonucleotides used in this study. (XLSX)
