# Supplementary figures and images for: Zebrafish rbm8a and magoh mutants reveal EJC developmental functions and new 3′UTR intron-containing NMD targets
Source: PLoS Genet. 2020 Jun 5;16(6):e1008830. doi: 10.1371/journal.pgen.1008830 (PMC7310861; doi:10.1371/journal.pgen.1008830)

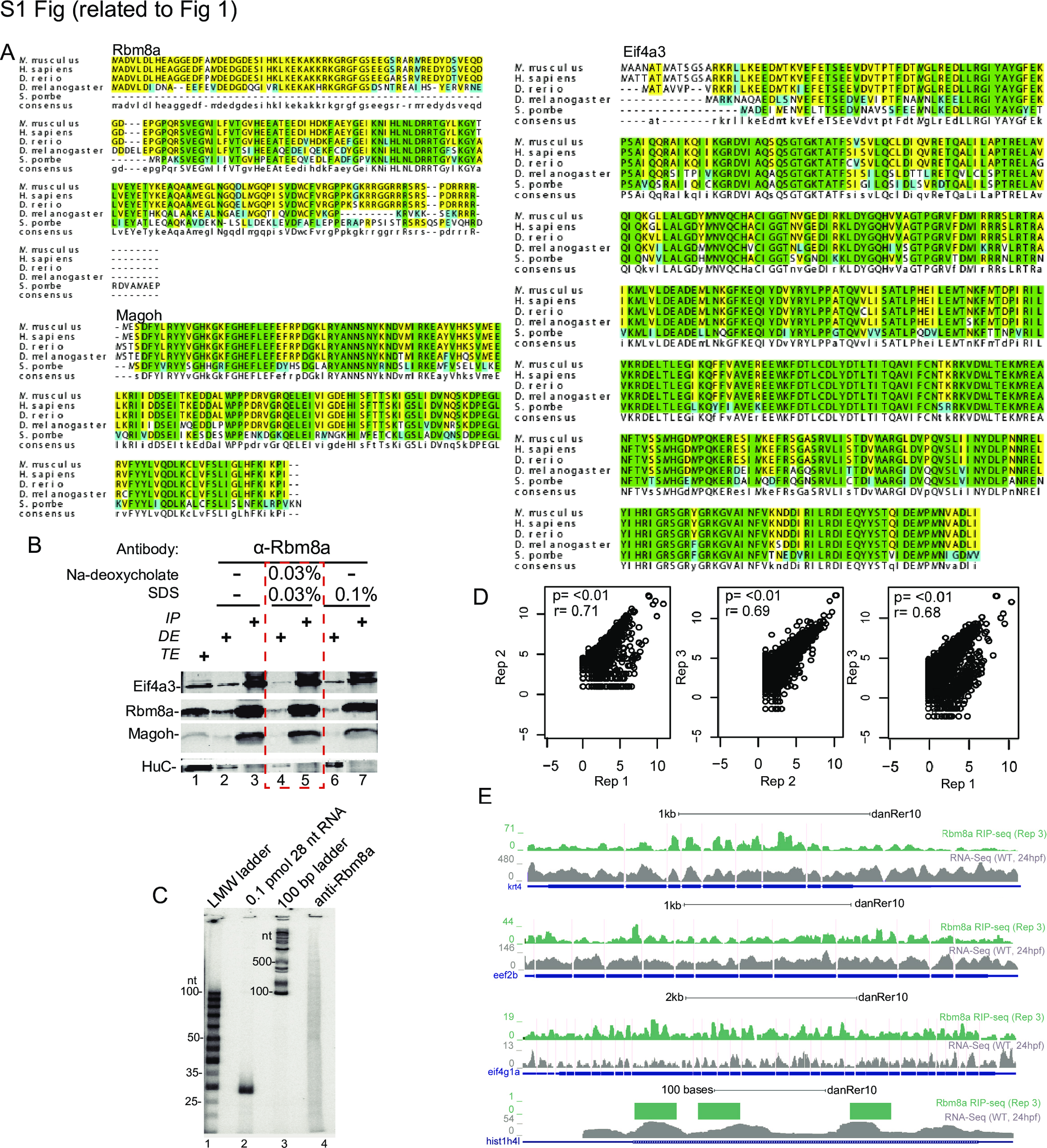

Supplement: S1 Fig — A. Multiple sequence alignments of Eif4a3, Rbm8a and Magoh protein sequences from organisms on the left. Consensus sequence is at the bottom with upper case letters indicating identity and lower case letters indicating similarity. Green indicates complete identity across all species, yellow and blue indicate the identical and unique amino acids in the regions with similarity. Identity between human and zebrafish EJC proteins: Eif4a3 (97%), Rbm8a (93%) and Magoh (100%). B. Western blot detecting proteins listed on the left in RNase I-treated zebrafish embryo total extract (TE, lane 1), depleted extract (DE, lanes 2, 4 and 6) and immunoprecipitates (IP, lanes 3, 5 and 7) with the Rbm8a antibody. Detergents supplemented to increase IP stringency are indicated on top of each lane. Optimized IP condition used in S1C is indicated by the dashed red box. C. Autoradiogram of γ32P 5′-end labeled RNAs from anti-Rbm8a RIP elution (lane 4) as well as indicated size-markers which include the low-molecular weight single-stranded DNA ladder (lane 1), 0.1 pmol 28 nt synthetic RNA (lane 2) and 100 bp DNA ladder (lane 3). D. Scatter plots comparing read counts for each gene in a pair of RIP-Seq replicates. The replicates (Rep1, Rep2, and Rep3) are indicated on the x- and y-axes. A pseudocount of 0.0001 was added to all genic read counts before log2 transformation. Pearson correlation coefficient (r) and p-value for the correlation test for each comparison is on the top left of each plot. E. Genome browser screenshots showing read coverage of Rbm8a RIP-Seq (only Rep 3, the deepest replicate is shown) in green and RNA-Seq in gray of select highly-expressed genes, krt4, eef2b, eif4g1a, and hist1h4l (intron-less gene). (TIF) [file pgen.1008830.s001.tif]

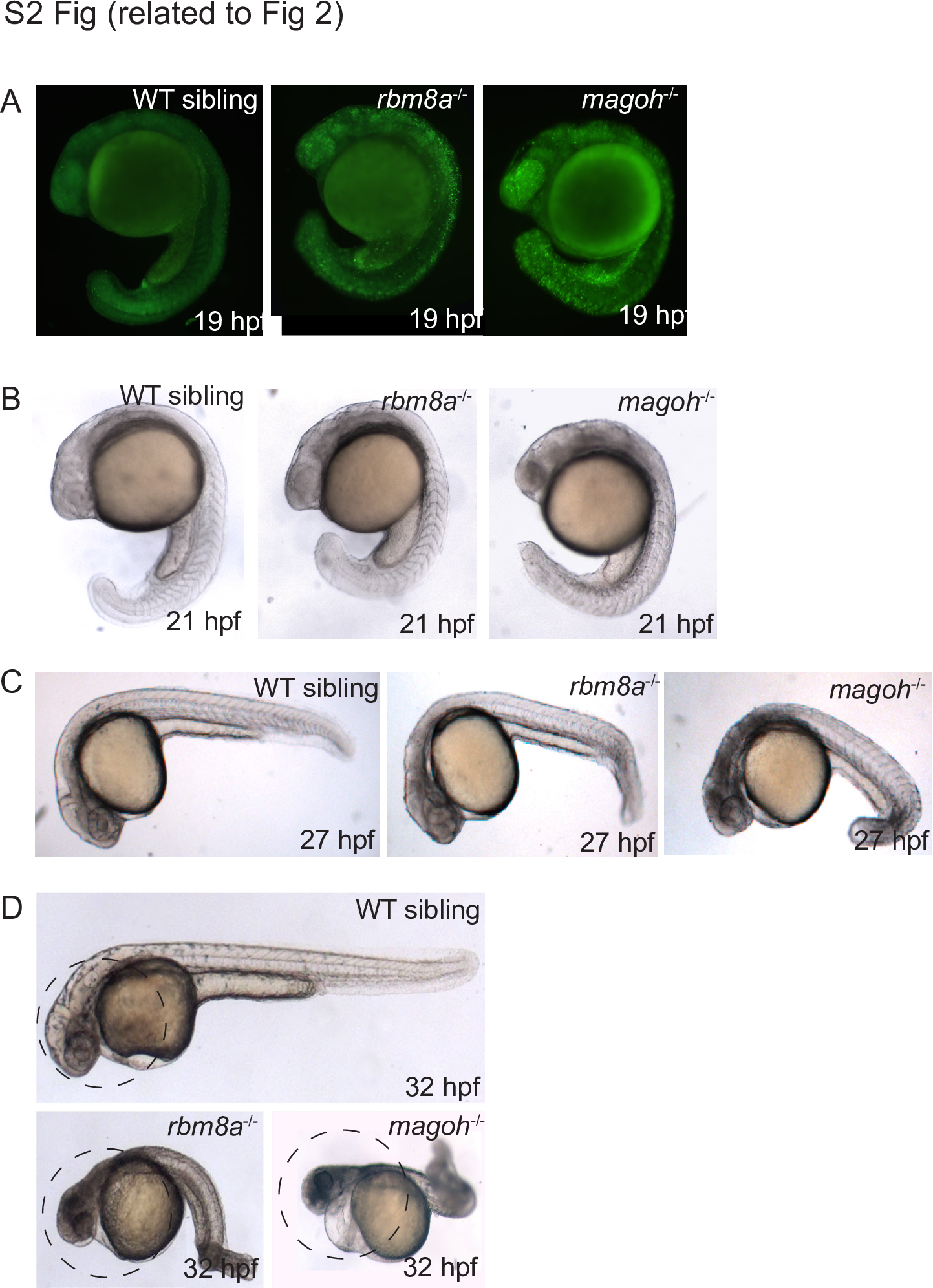

Supplement: S2 Fig — A. Whole mount images of live 19 hpf EJC mutant embryos and WT sibling embryos stained with acridine orange. B. Whole mount images of live EJC mutant embryos and WT siblings at 21 hpf. C. Whole mount images of live EJC mutant embryos and WT siblings at 27 hpf. D. Whole mount images of live EJC mutant embryos and WT siblings at 32 hpf. Head necrosis is indicated by the dashed circle. (TIF) [file pgen.1008830.s002.tif]

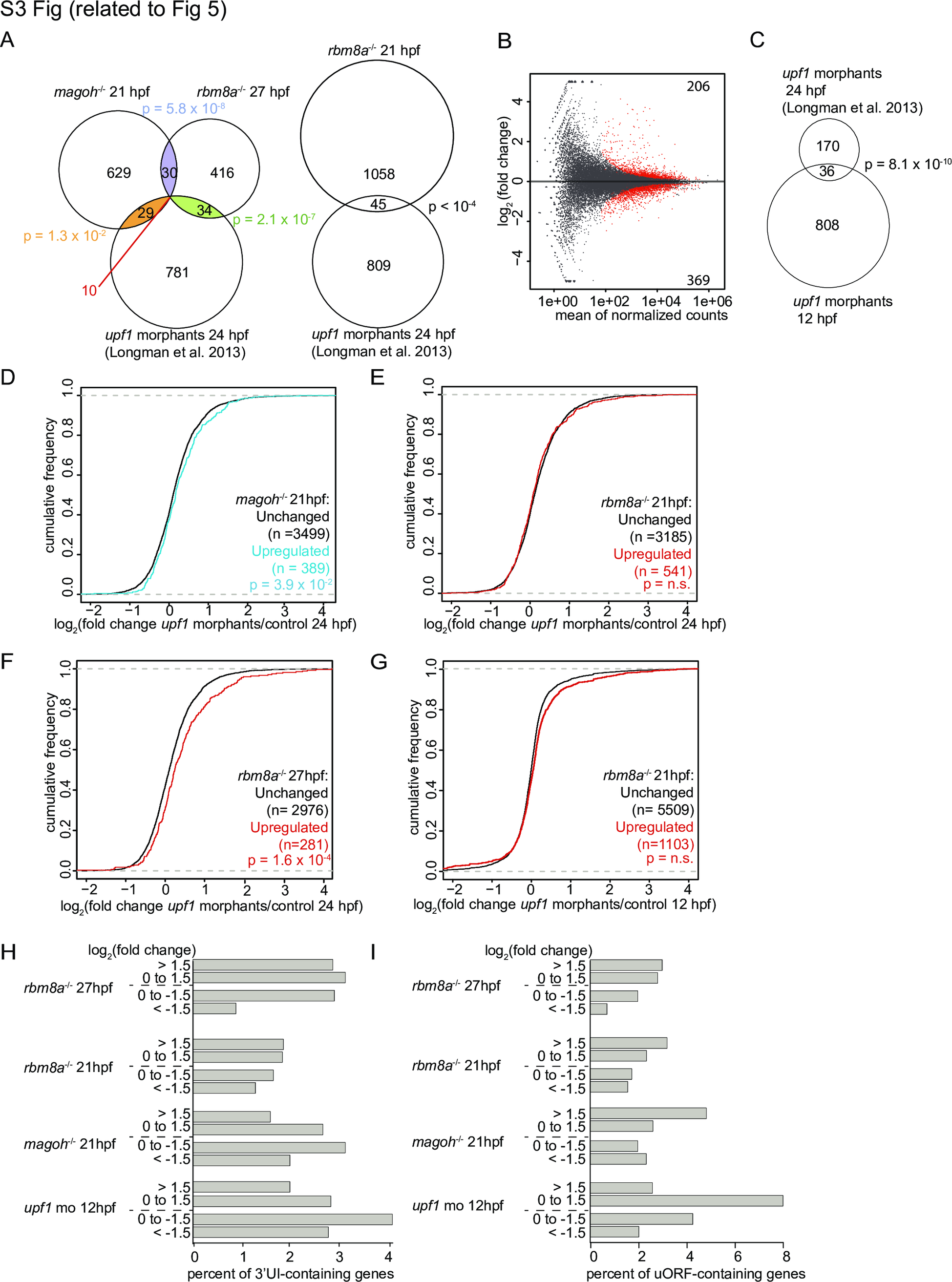

Supplement: S3 Fig — A. Venn diagram showing the overlap of genes that are significantly upregulated in EJC mutant embryos and upf1 morphant embryos at 24 hpf [55]. Hypergeometric test p-values for each comparison are also shown. B. MA plot showing the genes that are altered in expression (fold change > 1.5 and FDR < 0.05 in red and unchanged genes in gray) in upf1 morphant embryos compared to control embryos at 12 hpf. The number of significantly upregulated genes is at the top right and the number of downregulated genes is at the bottom right. C. Venn diagram showing the overlap of significantly upregulated genes in upf1 morphant embryos at 24 hpf [55] and upf1 morphant embryos at 12 hpf. Hypergeometric test p-value for the comparison is indicated. D. Empirical CDF plot showing the fold changes in upf1 morphant embryos (24 hpf) [55] of upregulated (blue) and unchanged genes (black) in 21 hpf magoh mutant embryos. Kolmogorov-Smirnov (KS) test p-value for differences in the two distributions are indicated at the bottom of the class descriptions. E. Empirical CDF plot as in S3D for upregulated (red) and unchanged genes (black) in rbm8a mutant embryos at 21 hpf. F. Empirical CDF plot as in S3D for upregulated (red) and unchanged genes (black) in rbm8a mutant embryos at 27 hpf. G. Empirical CDF plot showing the fold changes in upf1 morphants (12 hpf) of upregulated (red) and unchanged genes (black) in rbm8a mutant embryos at 21 hpf. H and I. Proportion of uORF-containing (H) or 3′UI-containing (I) genes in the total number of genes showing significant (FDR < 0.05) fold changes in rbm8a mutant, magoh mutant and upf1 morphant embryos. Genes are divided into four categories based on their log2 fold change: >1.5, 1.5 to 0, 0 to -1.5 and < 1.5. (TIF) [file pgen.1008830.s003.tif]

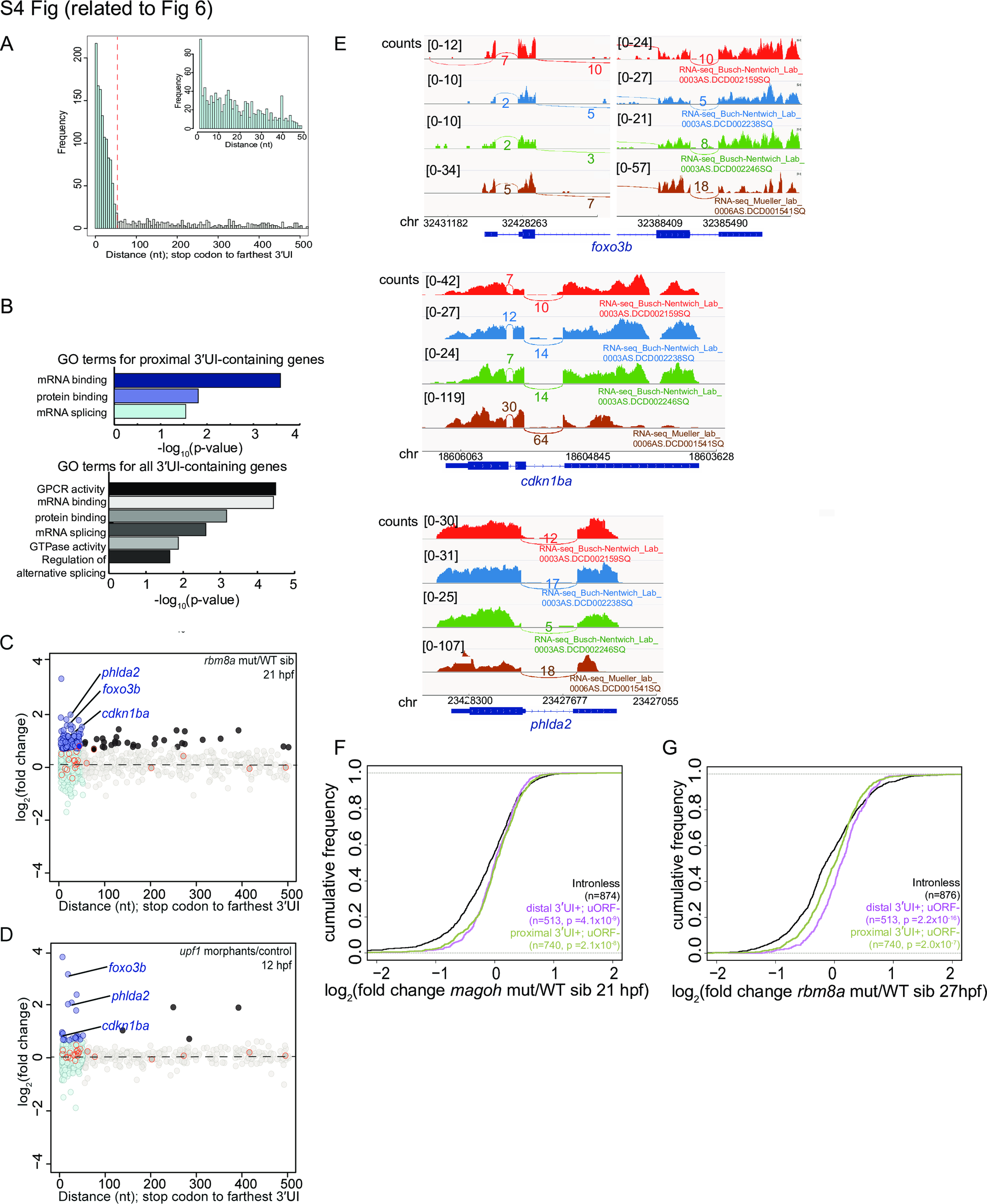

Supplement: S4 Fig — A. Histogram depicting the frequency of all zebrafish 3′UI transcripts in Ensembl GRCz10 (with APPRIS annotation) as a measure of the distance of the 3′UI from the stop codon. Data are shown in 5 nts bins and bins beyond 500 nts are not shown. Bins of proximal 3′UI genes are in blue and distal 3′UI bins are in gray. Inset: Histogram of all zebrafish proximal 3′UI transcripts binned by 1 nt. B. PANTHER14.0 [87] gene ontology (GO) term enrichment analysis of proximal 3′UI-containing genes (top, shades of blue) and all 3′UI-containing genes (bottom, shades of gray). All significant terms (Benjamini-Hochberg corrected p-value < 0.05) are shown for each set. C. A scatter plot showing gene-level fold change (FC) for transcripts with proximal 3′UI (dark blue: FC > 1.5 and light blue: FC < 1.5) and distal 3′UI (black: FC > 1.5 and gray: FC < 1.5) in rbm8a mutant embryos at 21 hpf compared to wild-type siblings. Genes encircled in red also contain a uORF as determined from a previously published dataset (see Materials and Methods). D. A scatter plot as in C showing fold changes of 3′UI-containing genes for 12 hpf upf1 morphants compared to wild-type control embryos. E. Integrated genome browser (IGV) screenshots of Sashimi plots showing RNA-seq reads observed for foxo3b, phlda2 and cdkn1ba in four zebrafish 24 hpf whole embryo RNA-seq datasets (as labeled on figure in different colors) obtained from the DanioCode consortium. Range of the number of reads mapping to the genes are indicated to the left of each track in black. Number of junction reads are indicated at the spliced junction in the color corresponding to the specific track. In case of foxo3b, due to the length of the second intron the screenshots of the first two and the last two exons are shown separately. F. Empirical CDF plot showing the fold changes in magoh mutants (21 hpf) of genes upregulated that contain a proximal (green) or distal (light purple) 3′UI or intron-less genes (black). Genes that also contained [file pgen.1008830.s004.tif]

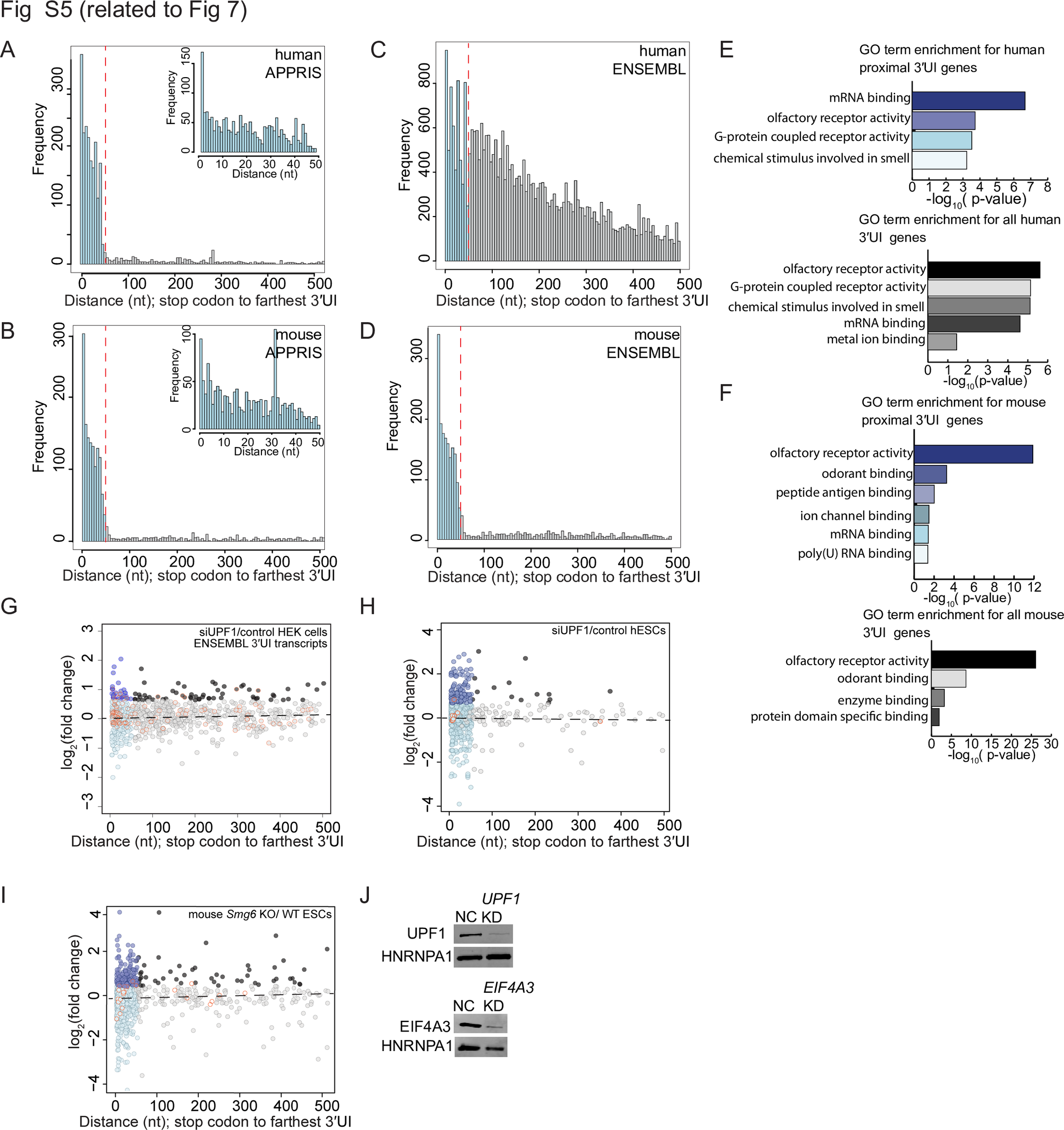

Supplement: S5 Fig — A. Histogram showing the frequency of all APPRIS 3′UI-containing transcripts in human GRCh38 as a measure of the distance of the 3′UI from the stop codon. Data are grouped in 5 nt bins from 1–500 nts. Proximal 3′UI-containing gene bins are indicated in blue; distal 3′UI-containing gene bins are indicated in gray. Red dotted line indicates distance from stop codon to farthest 3′UI = 50 nts. Histogram of transcripts from Ensembl annotation is shown in C. B. Histogram as in A of mouse proximal and distal 3′UI-containing transcripts in mouse GRCm38. Histogram of transcripts from Ensembl annotation is shown in D. C. Histogram as in A of proximal and distal 3′UI-containing human transcripts from Ensembl annotation of GRCh38. D. Histogram as in A of proximal and distal 3′UI-containing mouse transcripts from Ensembl annotation of GRCm38. E. PANTHER14.0 [86] gene ontology (GO) term enrichment analysis of human APPRIS proximal 3′UI-containing genes (shades of blue) and all human APPRIS 3′UI-containing genes (shades of gray). All significant terms (Benjamini-Hochberg corrected p-value < 0.05) are shown for each set. F. GO term enrichment analysis as in E of mouse APPRIS 3′UI-containing genes. G. A scatter plot showing gene-level fold changes for all Ensembl-annotated transcripts with proximal 3′UI (dark blue: FC > 1.5 and light blue: FC < 1.5) and distal 3′UI (black: FC > 1.5 and gray: FC < 1.5) in UPF1 knockdown human embryonic kidney cells (HEK293) compared to control cells using previously published data [57]. Genes encircled in red also contain a uORF as determined from a previously published dataset (see Methods). H. A scatter plot showing gene-level fold changes for all APPRIS-annotated transcripts with proximal 3′UI (dark blue: FC > 1.5 and light blue: FC < 1.5) and distal 3′UI (black: FC > 1.5 and gray: FC < 1.5) in UPF1 knockdown human embryonic stem cells (hESCs) compared to control cells using previously published data [62]. Genes encircled on red also contain a uOR [file pgen.1008830.s005.tif]

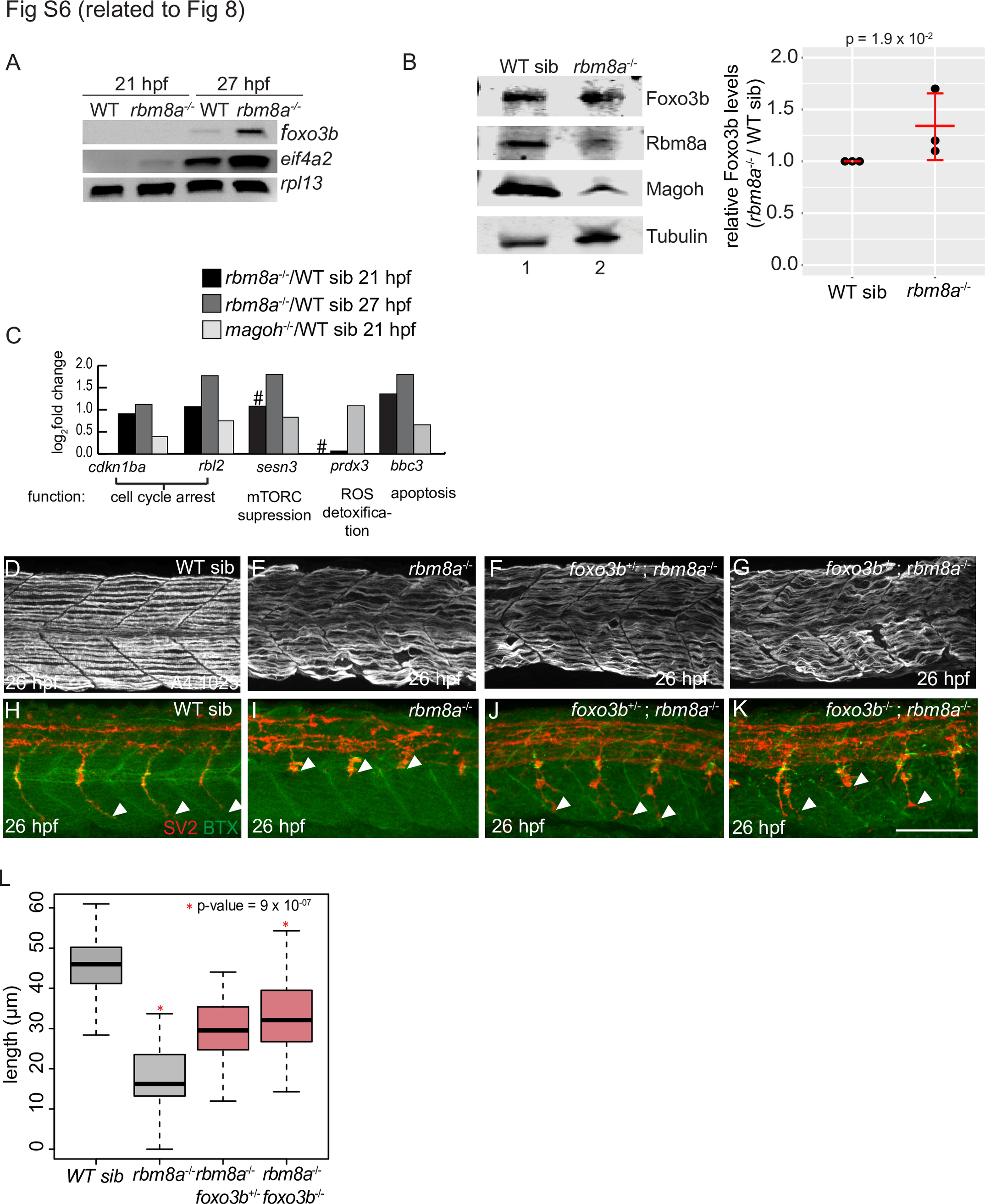

Supplement: S6 Fig — A. Semi-quantitative RT-PCR shows transcript levels of foxo3b, eif4a2 and rpl13 (loading control) in rbm8a mutant and wild-type sibling embryos at 21 and 27 hpf. B. Western blots (on the left) show levels of Foxo3b, Rbm8a, Magoh, and Tubulin in rbm8a mutant embryos (lane 2) compared to WT siblings (lane 1) at 27 hpf (N = 20 embryos per genotype). Right: dot plot showing Foxo3b levels normalized to tubulin levels in rbm8a mutant embryos and WT siblings at 27 hpf in three biological replicates. Error bars: standard error of means. Welch’s t-test p-values are indicated at the top. C. Bar graph showing log2 fold changes of known Foxo3b transcriptional targets that show a significant upregulation (FDR < 0.05) in EJC mutant RNA-Seq datasets. Foxo3b targets are from Morris et al. 2015 [64]. A pound symbol indicates statistically non-significant log2 fold change with FDR > 0.05. D-G. Confocal images showing Myh1 immunofluorescence using anti-A4.1025 in somites 12–16 of WT sibling (D), rbm8a-/- mutant (E), rbm8a-/-; foxo3b+/- mutant (F), and rbm8a-/-; foxo3b-/- mutant (G) embryos. (N = 5 embryos/genotype). H-K. Merged confocal images showing motor neurons (red; detected by anti-SV2 staining) and acetylcholine receptors (green; detected by alpha-bungarotoxin staining) in somites 12–16 of WT sibling (H), rbm8a-/- mutant (I), rbm8a-/-; foxo3b+/- mutant (J), and rbm8a-/-; foxo3b-/- mutant (K) embryos. Neuromuscular junctions in the merged image are yellow. White arrowheads point to the distal end of the motor neuron. (N = 5 embryos/genotype). Scalebar in K (for panels D-K) is 100 nm. L. Boxplots showing quantification of motor axon length in embryos of genotypes indicated along the x-axis) (4 motor neurons/embryo and 5 embryos/genotype). Welch’s t-test p-values are indicated at the top. (TIF) [file pgen.1008830.s006.tif]
